# A novel approach to estimate the impact of health workforce investments on health outcomes through increased coverage of HIV, TB and malaria services

**DOI:** 10.1186/s12960-023-00854-0

**Published:** 2023-08-21

**Authors:** Tim A. Bruckner, Tracy K. Lin, Jenny Liu, Olga Bornemisza, Onyema Ajuebor, Khassoum Diallo, Giorgio Cometto

**Affiliations:** 1 Program in Public Health and Center for Population, Inequality, and Policy, University of California, Irvine, USA; 2 Institute for Health and Aging, Bixby Center for Global Reproductive Health, University of California, San Francisco. 409 Illinois St. 123J, San Francisco, CA 94158 USA; 3 Technical Advice and Partnerships Department, The Global Fund, Chem. du Pommier 40, Le Grand-Saconnex, 1218 Geneva, Switzerland; 4 Health Workforce Department, World Health Organization, 20 Avenue Appia, 1211 Geneva, Switzerland

## Abstract

**Background:**

Globally, HIV, TB and malaria account for an estimated three million deaths annually. The Global Fund partnered with the World Health Organization to assist countries with health workforce planning in these areas through the development of an integrated health workforce investment impact tool. Our study illustrates the development of a user-friendly tool (with two MS Excel calculator subcomponents) that computes associations between human resources for health (HRH) investment inputs and reduced morbidity and mortality from HIV, TB, and malaria via increased coverage of effective treatment services.

**Methods:**

We retrieved from the peer-reviewed literature quantitative estimates of the relation among HRH inputs and HRH employment and productivity. We converted these values to additional full-time-equivalent doctors, nurses and midwives (DNMs). We used log-linear regression to estimate the relation between DNMs and treatment service coverage outcomes for HIV, TB, and malaria. We then retrieved treatment effectiveness parameters from the literature to calculate lives saved due to expanded treatment coverage for HIV, TB, and malaria. After integrating these estimates into the tool, we piloted it in four countries.

**Results:**

In most countries with a considerable burden of HIV, TB, and malaria, the health workforce investments include a mix of pre-service education, full remuneration of new hires, various forms of incentives and in-service training. These investments were associated with elevated HIV, TB and malaria treatment service coverage and additional lives saved. The country case studies we developed in addition, indicate the feasibility and utility of the tool for a variety of international and local actors interested in HRH planning.

**Conclusions:**

The modelled estimates developed for illustrative purposes and tested through country case studies suggest that HRH investments result in lives saved across HIV, TB, and malaria. Furthermore, findings show that attainment of high targets of specific treatment coverage indicators would require a substantially greater health workforce than what is currently available in most LMICs. The open access tool can assist with future HRH planning efforts, particularly in LMICs.

**Supplementary Information:**

The online version contains supplementary material available at 10.1186/s12960-023-00854-0.

## Introduction

In 2000, the United Nations (UN) committed member states to a series of Millennium Development Goals (MDG) to achieve by 2015. The sixth goal of the MDG focused increased international attention on combating HIV, tuberculosis (TB), and malaria [1]. From 2000 to 2015, the massive international response to HIV, TB and malaria markedly reduced global incidence and mortality of these three conditions [2]. Researchers estimate that concerted efforts, including those arising from large initiatives, such as the Global Fund to Fight AIDS, Tuberculosis and Malaria, saved over 50 million lives [3–6]. Most of these reductions concentrate in low- and middle-income countries (LMIC). HIV, TB and malaria, however, continue to be among the leading causes of disease in LMICs and account for an estimated three million deaths annually. These conditions, therefore, demand continued attention, as indicated by target 3.3 of the UN’s Sustainable Development Goal agenda for the year 2030.

Given the long-standing international focus on HIV, TB, and malaria, now sustained into target 3.3 of the 2030 Sustainable Development Goals, the Global Fund (GF) partnered with the World Health Organization (WHO) to assist countries with health workforce planning to serve the above disease areas, and broader health systems programmes as applicable. This partnership coheres with the broader “*WHO Global Strategy on Human Resources for Health: Workforce 2030*” plan which encourages development partners and global health initiatives to leverage their support to health systems in countries to strengthen the health workforce.

One area identified as a major gap in workforce planning is the lack of a user-friendly tool to clearly translate investments made in the health workforce into quantifiable estimates of health benefits, in particular, due to reduced HIV, TB, and malaria burden. Each of these diseases has cost-effective interventions which can be delivered by health workers. For this reason, investments in health workers may plausibly increase the number of people receiving preventive, treatment or care services for these diseases. Increases in treatment coverage, in turn, would save lives. The creation of a user-friendly tool that connects various types of health workforce investments to lives saved may allow estimating the results of allocation of governmental and nongovernmental resources.

The overarching aim of our paper is to illustrate the feasibility of developing a user-friendly tool [7] (with two MS Excel calculator subcomponents) that computes associations between human resources for health (HRH) investment inputs and reduced morbidity and mortality from HIV, TB, and malaria. A secondary aim is also to highlight the potential of the tool in understanding the impact (in previously unexplored dimensions) of HRH investments. We use recent health workforce, treatment service coverage, and global burden data to estimate the relationship between workforce investments, treatment service coverage, and lives saved. Our analysis, moreover, builds upon well-established methods and reports which show positive relations between health workforce and treatment service coverage for a wide range of conditions [8–13]. Given the significant investments WHO and GF make in health workforce in LMICs, and the large burden of HIV, TB, and malaria in these countries, we focus our tool on the end-user in a typical LMIC setting, though it may be applied in resource-rich contexts where fitting. The key research questions guiding the analysis are (i.) does investment in HRH improve health outcomes through an improved coverage of HIV, TB and malaria services? (ii.) What are the health workforce requirements to allow a scale-up of coverage of HIV, TB and malaria services?

As part of the tool development process, the calculator was pilot-tested to check for feasibility of use in selected LMICs. Here, we report results from models estimating the relationships among workforce investments, treatment service coverage, and lives saved due to reduced HIV, TB, and malaria. Next, we provide country profiles of Zambia and Pakistan, resulting from the pilot of the tool. We conclude with a discussion of the various ways our tool could assist with HRH planning and evaluation.

## Methods

### Conceptual model

**Fig. 1:**
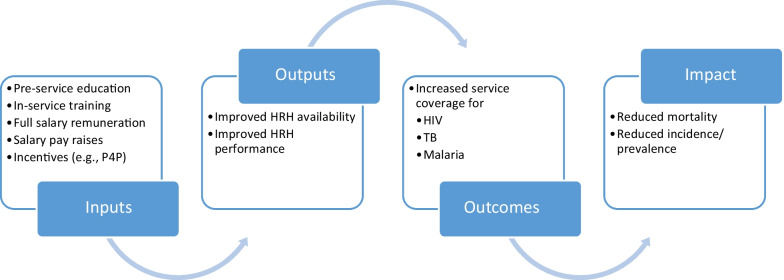
Conceptual model to assess health impact of HRH investment

Figure 1 provides the conceptual model by which HRH investments may translate into health impact in the areas of HIV, TB and malaria. HRH investments on the left box are considered inputs, which include investments in not only additional workers but also in productivity of current workers. These inputs improve the outputs of HRH availability and performance, which in turn translate into increase treatment coverage outcomes for HIV, TB, and malaria cases. When treatment rates increase for, say, antiretroviral therapy (ART) coverage for HIV patients, this improvement translates into a health impact of reduced HIV morbidity and mortality.

**Fig. 2:**
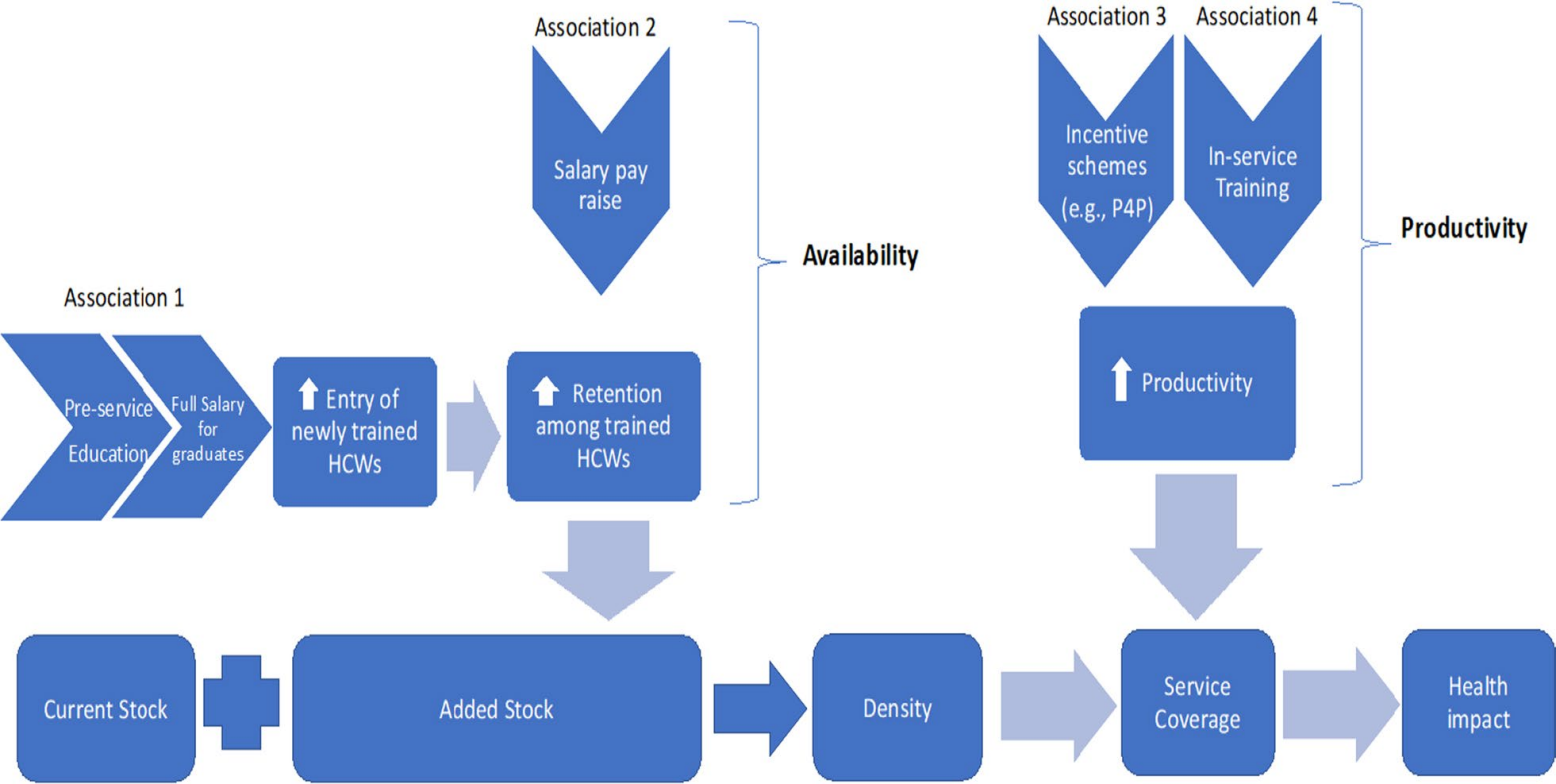
HRH investment pathways that affect HRH availability and performance

We focus on five specific types of HRH inputs (Fig. 2), These include pre-service education, full salary remuneration, increase in remuneration, incentive schemes, and in-service training. HRH inputs either add new workers or enhance productivity of current workers. The increased density of workers and improved performance, according to the conceptual model, both contribute to improved treatment service coverage and, ultimately, health impact.

### Analysis

To the extent that the arrows in the conceptual model capture true associations across the inputs, outputs, outcome and impact, a workforce tool which formalizes these associations would assist in HRH planning efforts. We, therefore, conducted an in-depth analysis to quantify these associations. We proceeded with the following steps. First, we retrieved from the most up-to-date peer-reviewed literature quantitative estimates of the relation among HRH inputs and HRH outputs. We converted these HRH outputs to additional full-time-equivalent (FTE) doctors, nurses and midwives (DNM). We specified these three occupational groups to be consistent with the literature estimating desired workforce density in LMICs [14] and for practical considerations, given that data availability for other occupational groups is limited. In addition, we included community health workers (CHW) and a category of health workers that is modifiable by end-user, with the default category being clinical officers (CO), in the end-user tool by applying a productivity multiplier empirically identified in the literature [15–19].

Second, we estimated the relation between DNM and treatment service coverage for HIV, TB, and malaria. Third, we retrieved treatment effectiveness parameters from the literature to calculate lives saved due to expanded treatment coverage for HIV, TB, and malaria. Below, we provide a brief overview of these three steps and refer the reader to Additional file 1 for the technical details explaining the model of the impact tool, and Additional file 2 for the technical details explaining the approaches to estimating the relationship between HRH density and service coverage.

#### Step 1: Translating investments into DNM employment and productivity

We focused our literature review on the five HRH investments indicated in Fig. 2: pre-service education, full salary remuneration, salary pay raises, incentives, and in-service training. Regarding pre-service education, we found no quantitative study that provided a global, systematic and generalizable estimate of the graduation rate among health education institutions in LMICs or the rate of employment of new graduates. We, therefore, set as a default in the tool a graduation rate of 80% for students enrolled in pre-service education programmes. As we acknowledge that graduation rates may vary dramatically across LMICs, the tool also allows end-users to input graduation rates specific to their settings. Among these graduates, the tool postulates that additional FTE salary support is needed to increase the number of graduates who go on to be fully employed as DNMs. Next, for the full salary remuneration input, increasing the employment of already-trained health workers in the health sector will add to the HRH workforce. We assumed that each new job employs one FTE DNM (assuming an 8-h workday).

Worker retention remains a key issue in HRH workforce planning. Increasing the salary of currently employed DNMs is one possible approach to retain health workers in their job [20]. One key study in LMICs by Rockers et al. [21] serves as the basis for estimating the additional salary needed to retain DNMs. This discrete choice experiment calculated the willingness-to-pay value that healthcare workers were willing to trade for other job characteristics. The willingness-to-pay value provides a monetary value of how much it is worth for a healthcare worker to stay or leave his/her current position, thus affecting the DNM labour supply. Results from the LMIC study indicate that a 54% increase in salary is necessary to retain 1 FTE DNM.

A comprehensive meta-review on the effects of interventions on performance of existing HRH workers, which includes financial incentives and in-service training, was conducted by Rowe et al. [22]. The authors derived a summary measure to aggregate measured effect sizes across 670 reports from 337 peer-reviewed publications, including 118 types of strategies, many of which included multiple intervention components. Results of the meta review indicated that, on average, financial incentives improved productivity by 1.2% points, whereas in-service training improved productivity by 11.5% points. Because the DNM productivity estimates were not specific to any disease, we assumed the same productivity values for DNM in improving HIV, TB, and malaria services.

#### Step 2: Relation between DNM and treatment service coverage

The relation between DNM concentration and HIV, TB, and malaria service coverage was estimated using the most recently, and publicly available DNM data to WHO at the time of our tests (i.e., through the WHO National health Workforce Accounts database hosted in its Global Health Observatory). These data include DNM density (i.e., DNM per 1000 population), with country-level UN Population Estimates [23] serving as the denominator.

Next, from various data sets archived in the World Bank’s Health Nutrition and Population Statistics database (https://datatopics.worldbank.org/health/), we identified 29 candidate treatment coverage indicators for HIV, TB, and malaria for which LMIC data are publicly available. Many of these coverage indicators were believed to relate to the conceptual framework, such that increases in DNM density in a country could plausibly lead to increases in the coverage rates of that treatment service. Based on this logic, candidate treatment indicators were examined using ordinary least-squares regression. This regression framework assumes a curvilinear, or “diminishing returns,” relation between DNM and treatment coverage. In separate regressions, we estimated the relation between each of the candidate indicators (dependent variables) and the natural logarithm of the number of DNM per 1000 population (independent variable). In the base specification, the number of observations was the total number of LMICs analyzed.

This analysis yielded four treatment service indicators with a positive and statistically detectable (i.e., *p* < 0.05) association with DNM concentration:

1. Antiretroviral therapy (ART) coverage (% of people living with HIV),
2. Percentage of pregnant women with HIV who receive ART to prevent mother-to-child transmission (PMTCT),
3. Tuberculosis treatment coverage: (number of new and relapse tuberculosis cases per number of incident cases); and
4. Percentage of children < 5 years with fever who sought treatment at any facility (an indicator of treatment for malaria)

Regression results for the four separate treatment service indicators appear in Table 1 (see Additional file 2 for technical details). The parameters for HIV, TB and malaria in the DNM coefficient column were used to estimate the gains in treatment service coverage associated with investments in DNM.

**Table 1:** Regression results predicting percent coverage of four separate treatment service indicators as a function of the natural logarithm of the number of DNM per 1000 population

Indicator	*N*^a^	DNM Coeff	SE	*p* value	Intercept
ART coverage for HIV	129	5.30	2.20	0.02	41.70
ART coverage for PMTCT	104	7.42	2.27	0.002	60.27
Tuberculosis treatment coverage	201	9.22	1.30	< 0.001	63.59
Children < 5 sought treatment for fever	70	6.05	1.8	0.002	61.86

^a^The number of countries analysed differed for each indicator owing to varying data availability for treatment service indicators across countries

#### Step 3: Lives saved due to expanded treatment service coverage

We reviewed the most up-to-date literature that estimates the effectiveness of the four HIV, TB, and malaria treatment services in LMICs (identified from the regression results showing positive associations with DNM density, in Table 1) in reducing mortality. Table 2 summarizes, by treatment indicator, estimates from the literature that we used to quantify lives saved in the calculator. We refer interested readers to the model of the impact tool [see Additional file 1] for technical details on the calculations in the table.

**Table 2:** Summary of “lives saved” estimation approach for HIV, TB, and malaria

Indicator	Lives saved	Country-level data from end-user	Key assumptions
ART coverage for HIV	5.11 lives saved per 100,000 population for each 1 percentage point increase in ART coverage	• Population size (optional: HIV prevalence)	• Effectiveness of ART coverage is similar to that of average effectiveness in Nigeria and South Africa
ART coverage for PMTCT	12.5 child deaths averted for every 100 additional HIV positive women on ART for PMTCT	• Percent of pregnant women who are HIV + that receive ART for PMTCT	• Effectiveness of ART coverage for PMTCT is similar to that of 21 sub-Saharan countries
Tuberculosis treatment coverage	1 life saved for every 3 TB cases treated	• Estimated number of all TB cases (detected) • TB treatment success rate	• TB treatment success rate remains fixed, regardless of level of TB treatment coverage
Children < 5 years sought treatment for fever	0.46% decline in count of < 5 malaria deaths for each percentage point increase in treatment-seeking	• Number of malaria-related deaths for under five children	• If a child seeks treatment for fever at a facility, they receive a diagnostic test for malaria

#### Benchmarking alternative

What DNM density is needed for target treatment coverage?

Based on conversations with end-users in LMICs, it became clear that users may benefit from an additional functionality of the tool which answers the question: how many additional DNMs would be needed in my country to attain a pre-identified “target” of treatment coverage? For example, this benchmarking alternative would permit the user to examine, for their country, the additional DNMs needed to attain the hypothetical treatment coverage target of 90% ART coverage for all persons with HIV. To arrive at this component of the calculator tool, we used observed DNM densities for countries with high percentages of treatment coverage and applied data envelopment analyses for countries with lower treatment coverage. Full methodologic details can be found in the approaches to estimating the relationship between HRH density and service coverage [see Additional file 2].

## Results

The synthesis of steps 1–3 appear in the “Lives saved” component of the calculator (MS Excel) tool in which the end-user enters HRH inputs and receives estimates of lives saved due to a reduced burden of HIV, TB, and malaria. We provide an example of the functionality of the “lives saved” component using a fictional country “*XYZ”* as well as an example of the “coverage target” benchmarking component of the tool. We then highlight two case-studies using actual HRH and epidemiologic data for Zambia and Pakistan [7].

To permit ease of following the fictional example, we will focus our country exercise on only one treatment indicator: antiretroviral therapy (ART) coverage for people living with HIV. “XYZ” is lower-middle income country with a population of 40 million, a current DNM density of 1.85 per 1000 population, a high HIV burden and an ART for HIV coverage of 64%. The following assumptions for five separate HRH investments relate to country “XYZ”.

- Investment 1: Assuming a cohort of 400 students, 90% of whom graduate per year, increase support for new graduate jobs by 80% leads to an additional density of 0.007.
- Investment 2: There is a 1:1 correspondence between the number of health workers hired and those added to the workforce.
- Investment 3: A 54% increase in remuneration leads to retaining 1 FTE DNM.
- Investment 4: Giving a remuneration increase leads to a performance increase of 1.2 percentage points.
- Investment 5: Giving in-service training leads to a performance increase of 11.5 percentage points.

Country “XYZ” plans to invest in a variety of areas. The country will increase the number of DNM graduates entering the labour market by supporting 80% more jobs for new graduates, increase salary pay by 10% for 10,000 DNMs, and provide in-service training to 10,000 CHWs. Table 3 provides the estimates of additional ART coverage for HIV and lives saved associated with these incremental HRH investments. Based on these inputs, ART coverage for HIV would increase from 64% to 64.092%, resulting in a corresponding 188 additional lives saved. If, alternatively, country “XYZ” used the “coverage target” component of the tool to estimate the number of additional DNMs needed to achieve 80% ART coverage, this estimate produces 120,800 additional DNMs needed. Another way to interpret this result is that, given the current 64% ART coverage and the current DNM density of 1.85 per 1000 persons, achieving 80% ART coverage would require an additional 2.71 DNM per 1000 persons.

**Table 3:** ART treatment coverage increase and HIV lives saved associated with a hypothetical set of HRH investments in country *XYZ*

HRH investment	Additional ART treatment coverage (percentage points)	Additional lives saved
Increase the number of graduates entering the labour market by supporting 80% more jobs for new graduates	0.01	20.4
Increase salary pay by 10% for 10,000 DNM	0.032	65.4
Give in-service training to 10,000 CHWs	0.05	102.2
Total	0.092	188

Selected case studies with actual HRH and epidemiologic data (as of 2019) **Zambia**

Zambia is a lower-middle-income country in south-central Africa with an estimated population of 16.6 million and DNM density of 0.54 per 1000 population. Zambia is challenged with a substantial burden of HIV, TB, and malaria. The number of pregnant women who received ART for prevention of mother-to-child transmission is 56,543 and TB cases are estimated at 62,000. Malaria is endemic and the number of malaria-related deaths is estimated at 7618.

The accountable workforce investments currently supported by the Global Fund in Zambia include pre-service training only for CHWs (*n* = 216), full remuneration of new hires (*n* = 557) and salary pay raises (*n* = 557). These investments span across the nursing and midwifery workforce, clinical officers, and community health workers. The data indicate that there was no investment made in medical doctors. The modelled estimates suggest that investments supported by the Global Fund resulted in 176 additional lives saved across the four treatment coverage areas, but the highest proportion of lives saved appears to have occurred through increased coverage of ART for HIV treatment.

If Zambia’s goal was to increase service coverage treatment by 5%, the additional DNM density needed would range from 0.45 to 2.42 DNMs per 1000 population depending on the specific treatment service provided.

**Pakistan**

Pakistan is a lower-middle-income country in the WHO Eastern Mediterranean Region. It has an estimated population of 189.4 million people and a DNM density of 0.32 per 1000 population. Pakistan faces the challenge of a substantial HIV, TB and malaria burden. The yearly number of pregnant women who receive ART for PMTCT is 532, and (notified) TB cases are estimated at 334,742. Malaria is endemic, and the number of malaria-related deaths is estimated at 138 per annum.

The accountable workforce investments currently supported by the Global Fund in Pakistan include pre-service training for CHWs (*n* = 51), doctors (*n* = 25), nurses and midwives (*n* = 20), full remuneration of new hires (*n* = 254) and salary pay raises (*n* = 1844). These investments span across medical personnel, the nursing and midwifery workforce, support personnel and CHWs. The modelled estimates suggest that HRH investments supported by the Global Fund resulted in approximately 367 additional lives saved across the four treatment coverage areas. The highest proportion of lives saved appear to have occurred through increased coverage of ART for HIV treatment. Pakistan has a goal of increasing treatment service coverage by 9%, 28%, 23% and 13% for ART for HIV, and ART for PMTCT, TB and children under 5 seeking treatment for fever, respectively. The additional DNM density needed will range from 0.09 to 7.03 DNMs per 1000 population depending on the specific treatment provided for the aforementioned service areas.

## Discussion

Governmental and non-governmental organizations continue to prioritize efforts to reduce the global burden of disease, and HIV, TB and malaria have received dedicated attention for over two decades. To assist stakeholders in LMICs with HRH workforce planning to combat these diseases, we developed an open access, user-friendly calculator tool featuring two MS Excel components—“Lives saved” and “Coverage target”. The tool demonstrates the feasibility of estimating improvements in service treatment and lives saved for HIV, TB and malaria following investments in the HRH workforce. Our review of the literature, as well as the empirical results of our novel analytic methodology, indicate positive associations among HRH investments, four key treatment service indicators, and health impact on the burden of HIV, TB, and malaria. In addition, pilot tests from LMICs, reported here and elsewhere [7], confirm the feasibility of using the tool to assist with HRH planning.

In most countries with a considerable burden of HIV, TB, and malaria, the health workforce investments supported in the context of programmes funded by global health initiatives such as the Global Fund include a mix of pre-service training, full remuneration of new hires, various forms of incentives and in-service training. These investments span across DNMs and CHWs. The investments, based on five pilot studies (which are reported here and in a separate publication [7]), typically occurred among CHWs. The modelled estimates developed for illustrative purposes and through country case studies suggest that HRH investments result in lives saved across the four treatment coverage areas. Furthermore, they show that attainment of high targets of specific treatment coverage indicators would require a substantially greater health workforce than what is currently available in most LMICs. Whereas investment in existing workers to improve their competencies and productivity is one useful approach, increasing the total number of HRH workers in these contexts represents a crucial long-term effort to achieve reductions in the burden of HIV, TB, and malaria, echoing predictions elsewhere [24]. Government strategies to increase the education and employment of the health workforce should provide the policy and investment framework to operationalize the support provided by development partners. In addition, we welcome partnering with other LMICs to further pilot-test, refine, and augment the model in future iterations.

The applicability of the workforce tool depends on availability of HRH output data as well as epidemiologic data. This information would include the country’s number of new health workers entering the workforce and the existing number of health workers trained, remunerated, incentivized or otherwise supported. Programmes and development partners, such as the Global Fund, that make a substantial investment in HRH should consider requiring the reporting of LMIC-specific information on these investments. Such required reporting would permit better tracking of “value added” from HRH investments and enable more precise estimates of the relation between HRH inputs and specific treatment service outputs.

Strengths of the tool include its user-friendly nature, in that our country counterparts for the piloting exercise could navigate and understand the various HRH and global burden data inputs. In addition, the most up-to-date literature and our empirical findings support positive associations between HRH inputs and health impact for HIV, TB, and malaria. Whereas components of the conceptual model have been applied in previous exercises to develop general benchmarks of DNM densities [25–27], we know of no prior effort which permits tailoring HRH evaluation to the particular country context. The current tool, moreover, gives the end-user substantial flexibility in entering various HRH investment scenarios, such that they could compare the influence of these scenarios on HIV, TB, and malaria treatment coverage.

A key limitation involves the descriptive nature of the associations discovered in our regression results. We used cross-sectional information to estimate the associations between country-level DNM density and treatment service coverage for HIV, TB, and malaria. The estimation strategy does not account for potential “third” variables which could cause both gains in DNM density and health. We, therefore, caution against interpreting as causal, the associations which underpin the HRH tool.

We also acknowledge a detailed set of simplifying assumptions (see Additional file 1). For instance, we assumed that new HRH investments do not affect existing HRH dynamics (e.g., inflows from immigration, outflows from departures or retirements). In addition, we applied the co-treatment rate for HIV/TB co-infected individuals, averaged across all countries, when estimating treatment service coverage following increased HRH investments. This averaging may mask important country-level differences in the extent to which LMICs aggressively screen for TB among HIV-positive persons. Whereas we make explicit these assumptions, the end-user should avoid interpreting any cell in the MS Excel calculator sheets as “deterministic,” precise estimates.

Regarding uncertainty analyses, our methodology assumes that the relations between the many health worker inputs and treatment service outputs are measured with no uncertainty. This simplifying assumption permits calculation of point estimates in terms of additional lives saved, for example, but does not provide confidence bounds for each point estimate.

Finally, while this tool does factor in different types of HRH investments and different occupational groups, it is not an allocative efficiency tool. For this reason, the tool cannot be used as a guide on which type of HRH investment or which occupational group (based on a marginal investment logic) should be prioritized. We, moreover, note that our estimates should in no way be construed as advocating for substituting HRH investments to treat HIV, TB and malaria, over general service staff in primary care settings to treat these and other conditions. We, rather, view the calculator tool as making the case about the value-added of estimating the results and impact of the HRH investments.

Investments in human resources for health have the potential for positive results for a range of health services and health outcomes, beyond the three diseases we examined. Future research may explore the application of our methodology to other health service areas. It is also acknowledged that HRH investments have broader positive development outcomes, including through the creation of qualified employment opportunities, particularly for women, spurring sustainable economic growth and contributing to gender empowerment. These other dimensions are of critical importance and deserve further scholarly attention.

### Supplementary Information

**Additional file 1.** Model of the impact tool and key assumptions of the calculator.

**Additional file 2.** Approaches to estimating the relationship between human resources for health (HRH) density and service coverage.

## Data Availability

Data used for the development of the investment impact tool are publicly available and are included in the respective sheets of the MS Excel calculators. Country data provided for the case studies are partially publicly available with the remaining only upon reasonable request.

## References

[1] United Nations Millennium Declaration. New York: United Nations,2000. (United Nations General Assembly Resolution 55/2. http://www.un.org/millennium/declaration/ares552e.pdf. Accessed 8 Jul 2004.

[2] Murray CJ, Ortblad KF, Guinovart C, Lim SS, Wolock TM, Roberts DA, Dansereau EA, Graetz N, Barber RM, Brown JC, Wang H (2014). Global, regional, and national incidence and mortality for HIV, tuberculosis, and malaria during 1990–2013: a systematic analysis for the Global Burden of Disease Study 2013. The Lancet.

[3] World Health Organization. Accelerating progress on HIV, tuberculosis, malaria, hepatitis and neglected tropical diseases: a new agenda for 2016–2030.

[4] World Health Organization. World malaria report 2015. World Health Organization; 2016 Jan 30.

[5] WHO, UNAIDS. HIV estimates with uncertainty bounds 1990–2014. Geneva: World Health Organization (WHO), Joint United Nations Programme on HIV/AIDS (UNAIDS); 2015 http://www.unaids.org/en/resources/documents/2015/HIV_estimates_with_uncertainty_bounds_1990-2014. Accessed 04 Jan 2016.

[6] WHO. Global tuberculosis report 2015. Geneva World Health Organization (WHO); 2015 http://www.who.int/tb/publications/global_report/gtbr15_maintext.pdf. Accessed 04 Jan 2016.

[7] WHO (2022). Tool to assess impact of human resources for health investments on HIV, TB and malaria services and health outcomes.

[8] Anand S, Bärnighausen T (2012). Health workers at the core of the health system: framework and research issues. Health Policy.

[9] Anand S, Bärnighausen T (2007). Health workers and vaccination coverage in developing countries: an econometric analysis. The Lancet.

[10] Castillo-Laborde C (2011). Human resources for health and burden of disease: an econometric approach. Hum Resour Health.

[11] McPake B, Maeda A, Araujo EC, Lemiere C, El Maghraby A, Cometto G (2013). Why do health labour market forces matter?. Bull World Health Organ.

[12] Robinson J, Wharrad H (2000). Invisible nursing: exploring health outcomes at a global level–relationships between infant and under-5 mortality rates and the distribution of health professionals, GNP per capita, and female literacy. J Adv Nurs.

[13] Scheil-Adlung X (2013). Health workforce benchmarks for universal health coverage and sustainable development. Bull World Health Organ.

[14] Bruckner T, Liu J, Scheffler RM. Demand-based and needs-based forecasts for health workers. Health labor market analyses in low-and middle-income countries. 2016; 8:49.

[15] Jafar TH, Islam M, Bux R, Poulter N, Hatcher J, Chaturvedi N, Ebrahim S, Cosgrove P (2011). Cost-effectiveness of community-based strategies for blood pressure control in a low-income developing country: findings from a cluster-randomized, factorial-controlled trial. Circulation.

[16] Dick J, Clarke M, Van Zyl H, Daniels K (2007). Primary health care nurses implement and evaluate a community outreach approach to health care in the South African agricultural sector. Int Nurs Rev.

[17] Seidman G, Atun R (2017). Does task shifting yield cost savings and improve efficiency for health systems? A systematic review of evidence from low-income and middle-income countries. Hum Resour Health.

[18] Vaughan K, Kok MC, Witter S, Dieleman M (2015). Costs and cost-effectiveness of community health workers: evidence from a literature review. Hum Resour Health.

[19] McCoy D, Bennett S, Witter S, Pond B, Baker B, Gow J, Chand S, Ensor T, McPake B (2008). Salaries and incomes of health workers in sub-Saharan Africa. The Lancet.

[20] Ajuebor O, Boniol M, McIsaac M, Onyedike C, Akl EA (2020). Increasing access to health workers in rural and remote areas: what do stakeholders' value and find feasible and acceptable?. Hum Resour Health.

[21] Rockers PC, Jaskiewicz W, Wurts L, Kruk ME, Mgomella GS, Ntalazi F, Tulenko K (2012). Preferences for working in rural clinics among trainee health professionals in Uganda: a discrete choice experiment. BMC Health Serv Res.

[22] Rowe AK, Rowe SY, Peters DH, Holloway KA, Chalker J, Ross-Degnan D (2018). Effectiveness of strategies to improve health-care provider practices in low-income and middle-income countries: a systematic review. Lancet Glob Health.

[23] United Nations Population Division. World population prospects: 2019 revision. New York: United Nations Population Division; 2019 https://population.un.org/wpp/. Accessed 19 Jul 2021.

[24] Liu J, Goryakin Y, Maeda A, Bruckner T, Scheffler R (2017). Global health workforce labor market projects for 2030. Hum Resour Health.

[25] World Health Organization. Health workforce requirements for universal health coverage and the Sustainable Development Goals. Human Resources for Health Observer, 17.

[26] Liu JX, Goryakin Y, Maeda A, Bruckner T, Scheffler R. Global health workforce labor market projections for 2030. The World Bank; 2016. 10.1186/s12960-017-0187-2 PMC5291995 28159017

[27] Scheffler RM, Campbell J, Cometto G, Maeda A, Liu J, Bruckner TA, Arnold DR, Evans T (2018). Forecasting imbalances in the global health labor market and devising policy responses. Hum Resour Health.

