# Supplementary material for: A novel approach to estimate the impact of health workforce investments on health outcomes through increased coverage of HIV, TB and malaria services
Source: Hum Resour Health. 2023 Aug 21;21:67. doi: 10.1186/s12960-023-00854-0 (PMC10441693; doi:10.1186/s12960-023-00854-0)
Supplement: Supplementary file 1 — Additional file 1. Model of the impact tool and key assumptions of the calculator. [file 12960_2023_854_MOESM1_ESM.pdf]

Model of the impact tool and key assumptions of the calculator

Steps 1 to 3 in the section below helps to illustrate in deeper details, the main features, evidence base and assumptions of the HRH impact assessment tool [1], linking investments to improved availability, distribution and performance of HRH.

Step 1. Investment in HRH through education, improved deployment, remuneration, availability, distribution, and performance

A. Investment 1: Pre-service education

It is assumed that any HRH investments occur above and beyond existing levels of investment; associations between investment options and HRH density or performance were based on the peer-reviewed literature. Few studies examined the association between the number of entrants in health education institutions and the number of graduates. No quantitative study was found that provided a systematic estimate of the graduation rate among health education institutions in LMICs or the rate of employment of new graduates.

A default graduation rate of 80% was set for students enrolled in pre-service education programmes. Among these graduates, the model postulates that additional full-time employment salary support (as compared to pre-existing employment levels) is needed to increase the number of graduates who go on to be fully employed as health workers (above and beyond steady-state inflows); the tool allows modification of these parameters if context-specific empirical data are available.

B. Investment 2: Hiring

Simply increasing the employment of already-trained health workers in the health sector will add to the HRH workforce. It is assumed that each new job employs one FTE (assuming an 8-hour workday).

C. Investment 3: Salary raises

Increasing the salary of currently employed health workers is one possible approach, among others, to retain health workers. The base wage is excluded because the incremental increase is conceptualized to target currently employed workers. Hiring additional workers via full salary support is reflected in Investment 2.

Literature on the impact of wages on labour [2,3] includes quantitative analyses such as discrete choice experiments (DCEs).

Remuneration was included as an intervention in most of the DCE studies (*N* = 24). Most DCE studies in LMICs find that remuneration is a statistically significant factor for encouraging healthcare workers to relocate to, or remain at, specific locations that may otherwise have fewer desirable characteristics (see Table 2 below**)**. We examined published studies that provided an estimate translatable to several outcomes: (i) retention in the country, (ii) willingness to work in poorly equipped facilities,[1] (iii) remaining at a specific location (e.g., rural) or position and (iv) relocation to a rural/remote location. Retention of the workforce in the healthcare sector or the country was the most relevant outcome for estimating the effect of salary pay raises on HRH availability.

Many of the DCE studies calculated the willingness-to-pay (WTP) value that health workers were willing to trade for other job characteristics. In the context of HRH, the WTP value provides a monetary value of how much it is worth for healthcare workers to stay or leave their current position, thus affecting the labour supply. Using the reported WTP value, the WTP can be calculated as a percentage of workers' salary, which can be interpreted as the increase in resources needed to retain a healthcare worker among those in the study that took up the offer.

---

[1]In qualitative interviews conducted to outline attributes for DCEs, this outcome has been consistently outlined as a salient characteristic influencing individual decisions to leave or remain in a country.

Three studies (from LMICs that included doctor and/or nurse/midwife occupational groups) met the inclusion criteria [4,5,6]. However, only one of the three studies – i.e., Rockers et al. [5] – reported the fraction of study participants willing to take the pay raise, which enabled calculation of an estimate.

D. Investments 4 and 5: Incentives and in-service training

A recent meta-review on the effects of health worker interventions on HRH productivity was conducted by Rowe et al. (2018) [7]. This study is the most comprehensive review conducted to date that is directly relevant to the objective of obtaining elasticities related to performance outcomes.

Rowe et al. presented study results for improving health-care provider practice expressed either as percentages (e.g., percentage of patients treated correctly) or as continuous measures (e.g., number of medicines prescribed per patient). The summary measure to aggregate measured effect sizes across studies was the median effect size (MES). The MES values from the included studies were derived from 670 reports from 337 peer-reviewed publications, including 118 types of strategies, many of which included multiple intervention components.

In studies that focused on either financial incentives or training as the sole intervention, the MES calculated provides information on the intervention's impact on specific outcomes. Studies reviewed that describe the effect of financial incentives or in-service training on HRH performance (Investments 4 and 5, respectively) tended to evaluate incentive packages or policies which involved a combination of different intervention approaches. For example, Perez-Cuevas et al. [9] examined the effect of the interventions of group problem solving, supervision, other management techniques and training conjointly. The MESs reflect the change in performance outputs in response to the presence of any training or financial incentives. Table 1 summarizes these estimates and the number of studies reviewed on which they were based.

The information is from Rowe et al. (2018) [7] and the associated supplementary appendix, and Rowe et al. 2019 [9] and the Health Care Provider Performance Review online database [10].

Table 1. Studies examining the association between remuneration or training only, and performance

**Intervention**	**Number of studies**	**Percentage point difference**	**Median of MES values (expressed in percentage)**	**Interquartile range**	**Minimum**	**Maximum**
Training	82	**11.5**	8	3.1–18.4	–21.3	68.1
Financial incentives	2	**1.2**	25.9	N/A	11.1	40.7

Composite estimates in the column for **percentage point difference** were used for Investments 4 and 5. Proceeding with the aggregate MES, the following additional assumptions were made:

- Service coverage was defined broadly as improvement in service delivered; the variable is not disease-specific.
- Performance was defined as the increase in correct behaviour and treatments administered (or alternatively as the reduction in incorrect or inefficient behaviour and treatments administered).
- Any resulting performance improvement was linearly additive onto the HRH service coverage that results from HRH density.

Because service coverage is not specific to disease according to the empirical basis used to estimate these coefficients, the assumptions on increase in coverage adopted in the model are the same for HIV, TB, and malaria services.

E. Multiplier for community healthcare workers

Estimates of the empirical association between health worker density and service coverage for HIV/AIDS, TB and malaria are based on actual cross-country data on density of skilled health professionals, and specifically of the workforce of doctors, nurses and midwives. Cross-country data for community healthcare workers (CHWs), however, are less complete and thus insufficient to use to separately estimate an empirical relationship with service coverage. This was a notable limitation of the available data: future work could benefit from refinement of methods as more data on additional occupational groups become more widely available.

To estimate how HRH investments on CHWs can affect treatment service coverage and lives saved (and vice versa), a productivity multiplier was used, reflecting the ratio of the productivity of a doctor or nurse/midwife to CHW. A literature search identified studies that provided suitable estimates of this relative productivity (see later section of this supplementary file). As an example, one study found that when tasks were shared between DNMs and CHWs, compared to solely being performed by DNMs, the follow-up use of antihypertensive medication increased from 46.3% to 49.7% [11]. Another study found a similar change in the TB treatment completion rate from 64.3% to 83.0% when CHWs were added to the skills mix [12].

Two systematic literature reviews on cost–effectiveness analyses (CEAs) and optimal skills mix [13,14] were helpful in developing specific estimates for comparing changes in effectiveness of a team of CHWs and DNMs, as compared to DNMs providing care alone. The changes in

effectiveness between the two groups were then translated into changes in overall productivity for each intervention, allowing calculation of a productivity multiplier for converting DNM to CHW equivalents.

Four CEA estimates were extracted from the literature which enabled conversion of DNM productivity into CHW productivity (see MS Excel sheet "X. CHW_calc" in the Lives Saved and Coverage Target calculators and later section of this supplementary file). The multiplier was calculated by taking the change in service coverage percentage points given the involvement of CHWs, and assuming that the change is due to the increase or decrease in productivity of CHWs compared to DNMs [15].[2] The descriptive statistics for the multipliers are included in Table 2 below (individual study estimates are provided in Table 3 in the later section of this supplementary file). Given the range of estimates, the median of the productivity ratios was used as the multiplier.

Table 2. Descriptive statistics for the ratio of productivity of DNMs to CHWs

Observations	Median	Min	Mean	SD	Max
4	**1.1065**	1.020	1.105	0.090	1.187

SD: standard deviation.

[2] For example, for estimates from Okello and colleagues the productivity multiplier was calculated by dividing the TB treatment success for DNMs (74%) by that when CHWs were added to the skills mix (56%), which yields the multiplier of 1.32.

Step 2. Relationship between human resources for health (HRH) density and increased coverage of selected services related to HIV, TB and malaria,

A. Primary analysis of service coverage indicators

Eleven of the 29 candidate indicators were examined using ordinary least squares (OLS) regression (see “”Z.MD_only” and “Z.NM_only” tab in Lives saved calculator: tool”) [1]. This framework assumes a curvilinear, or “diminishing returns”, relationship between DNM and HIV, TB, and malaria treatment coverage. In this approach, the greatest increases in treatment service coverage are associated with initial investments in DNM. At a certain level of DNM, however, there is an inflection point at which the rate of increase in treatment service coverage is smaller with each additional DNM investment.

First, the relationship between each of the 11 candidate indicators (dependent variables) and the natural logarithm of the number of DNMs per 1000 population (independent variable) was estimated. In the base specification, the number of observations was the total number of LMICs analysed. This analysis yielded four treatment service indicators with a positive and statistically detectable (i.e., $P < 0.05$) association with DNM concentration (see Table 3 in this document):

- Antiretroviral therapy (ART) coverage (% of people living with HIV);
- percentage of pregnant women with HIV who receive antiretroviral medicine for prevention of mother-to-child transmission (PMTCT);
- TB treatment coverage: the number of new and relapse TB cases per number of incident cases; and
- the percentage of children < 5 years with fever who sought treatment at any facility (a proxy indicator of treatment for malaria).

Each treatment service indicator serves as a separate dependent variable in a separate regression. When each of the four regressions was restricted to LMICs only, all but the first HIV indicator (ART coverage for people living with HIV) showed a positive and statistically detectable association. However, when using the full country data set without any income-based country restrictions, all four indicators – two for HIV, one for TB and one for malaria – showed a positive and statistically detectable association. The full-country results of each of the four OLS regression analyses appear in Table 3 below. Note that the number of countries analysed differed for each indicator, owing to varying data availability for treatment service indicators across countries.

Table 3. Four separate regression results for four treatment service indicators

**Indicator**	**No. of countries**	**DNM coefficient**	**SE**	***P* value**	**Intercept**
ART coverage for HIV	129	5.30	2.20	.02	41.70
ART coverage for PMTCT	104	7.42	2.27	.002	60.27
TB treatment coverage	201	9.22	1.30	< .001	63.59
Children < 5 sought treatment for fever	70	6.05	1.8	.002	61.86

SE: standard error.

**Each treatment service indicator serves as the dependent variable in that respective regression; the natural logarithm of DNMs per 1,000 population is the independent variable in each regression.*

These parameters allowed estimation of treatment service coverage for a particular DNM density. For example, for TB treatment coverage a few levels of DNM per 1,000 population,

corresponding to a specific level of treatment service coverage, are provided for purposes of illustration (Table 4 below).

Table 4. Sample levels of TB treatment service coverage by value of DNM concentration

DNM concentration per 1,000 population	Estimated TB treatment coverage
1.0	63.59
1.5	67.33
2.0	69.98
2.5	72.03
4.0	76.37

The regression equation results illustrated in Tables 3 and 4 above were then used to estimate the gains of treatment service coverage associated with investments in DNMs. Users may also identify lower and upper confidence bounds estimates of “lives saved” for additional coverage of each treatment service indicator. These confidence bounds use the standard errors of coefficient estimates (Table 3 above) to derive 95% confidence intervals of lives saved.

B. Benchmarking alternative

A complementary function of the model and calculator developed entails a possibility for the user to set a predetermined level of treatment service coverage for HIV, TB and malaria and then find the level of aggregate workforce requirements to attain that service coverage.

The benchmarking alternative entails use of empirically derived maximum “caps” as well as minimum “floors” (and the range in between the cap and floor) of general DNM concentrations that correspond with existing treatment service coverage levels. The maximum cap was derived

by identifying the median level of DNM observed for countries that meet the service coverage target for that particular indicator (e.g., 10.85 DNM for the 12 countries which exceeded 90% treatment coverage for TB). The minimum floor of DNM, by contrast, was created using results from a data envelopment analysis (DEA), which ranks countries according to their efficiency in delivering treatment service coverage per level of DNM. DEA is a statistical analysis tool often applied to microeconomics and operations management. In this context, the tool has been applied to identify countries that maximize the utility of existing resources to achieve a desired end. Specific details for the DEA approach are covered in Supplementary file 2.

Once the DNM maximum cap and floor levels for each treatment service indicator had been determined, a log-linear function was fitted through these points (given the economic principle of diminishing returns to additional DNM especially at higher levels of DNM).

C. Accounting for comorbidity

In countries with a relatively high incidence of HIV and TB, there is a dual burden from HIV/TB coinfection [16]. Despite the comorbidity associated with HIV/TB coinfection, many coinfected patients remain undetected and undertreated [17]. In light of this challenge, WHO outlined guidelines and policy recommendations for enhancing collaborative HIV/TB activities [18].

Based on empirical estimates available in the studies quoted in this subsection, estimates of HIV/TB coinfection and HIV/TB detection rates were integrated when estimating how HRH investments could augment treatment service coverage across these two conditions. In a study from a setting with a high HIV burden, among HIV-positive persons who had screened positive for TB at the time of the study, only 6.3% were receiving TB therapy [19]. This study underscores the sometimes low level of integration of HIV and TB control activities, but indicates that persons who test positive for HIV may also undergo additional TB screening. It is

therefore assumed that, in a high HIV/TB coinfection context, additional treatment of, say, 100 HIV-positive persons would increase TB screening for ~6.3 TB-positive persons (i.e., 6.3%).

Next, the increase in TB service treatment coverage attributable to an increase in HIV treatment coverage was calculated for countries with lower HIV/TB incidence. Estimates of HIV/TB incidence were taken from the Global Burden of Disease project [20]. It was assumed that countries with a lower HIV/TB coinfection rate would necessarily have a slightly lower "cross-benefit" for TB detection among persons undergoing HIV treatment. This cross-benefit in TB detection ranged from 3.3% to 6.3% and was automatically integrated into the final TB service treatment coverage percentage.

D. Interpretation

Table 3 provides estimates of the relation between DNM density and treatment service coverage for four treatment service coverage indicators referring to specific HIV, TB and malaria services. Given the instability of the HIV ART treatment indicator results in alternative approaches tried, which exclude high-income countries and have a relatively low sample size, we recognized that further stratification of our regression approaches by country characteristics (e.g., low-income countries – LICs – only) attenuates the DNM / treatment coverage associations or renders them nondetectable. The functionality of the lives saved calculator also includes options to cater to the specific aspects of a country's situation. For example, countries without endemic malaria may wish to incorporate an assumption that HRH investments are not related to malaria burden in that country. This context-specific aspect, as well as the "high or low HIV burden" button, can thus be clicked to activate these aspects into the modelling estimates.

Step 3. Translating service coverage into health impact

The OLS regression analysis described earlier yielded four treatment service coverage indicators for HIV, TB and malaria that show a positive association with DNM density:

- ART coverage (% of people living with HIV);
- percentage of pregnant women with HIV who receive antiretroviral medicine for prevention of PMTCT;
- TB treatment coverage: the number of new and relapse TB cases per number of incident cases; and
- the percentage of children < 5 years with fever who sought treatment at any facility (an indicator of treatment for malaria).

These four indicators were used to model how improvements in service coverage, via HRH investments, may translate into lives saved. Relevant empirical data from the literature were reviewed to derive estimates of lives saved.

A. Lives saved due to increased ART coverage

A study reported trends in HIV incidence, ART coverage and mortality statistics from the 30 countries with the greatest AIDS mortality burden [21]. Based on results using data from the Spectrum/EPP software AIDS Impact Module, estimates of lives saved per unit increase in ART coverage were derived. The manuscript reports results for two countries that account for 27% of the global HIV burden.

The first country reported a 20% ART coverage rate for persons with HIV. If this country were to maintain its current level of ART coverage, the researchers estimated 485,564 fewer AIDS-related deaths over a 7-year period from 2014 to 2020. This estimate equates to an average of 69,366 lives saved per year associated with 20% ART coverage. Given this estimate, a 1

percentage point increase in ART coverage could avert 3,468 deaths per year (i.e., 69,366 / 20). If these lives saved were scaled to the population size of that country, a 1% increase in ART coverage would correspond to 1.89 lives saved per 100,000 population.

If, instead, we used figures for the second country that was generated by the AIDS Impact Module, the reported ART coverage of 42% would correspond to a projected 1,363,201 fewer AIDS-related deaths from 2014 to 2020 [21]. This projection equates to an average of 194,743 lives saved per year. A 1% increase in ART coverage, therefore, could avert 4,637 deaths (i.e., 194,743 / 42). If these lives saved estimates were scaled to the population size of the second country, a 1% increase in ART coverage would thus correspond to 8.33 lives saved per 100,000 population.

If we take the average number of AIDS-related lives saved per 100,000 population (in the first and second country) for a 1 percentage point increase in ART coverage, which assumes additivity of health benefits per unit increase in treatment coverage given low HIV incidence:

(1.89 + 8.33) / 2 = 5.11 lives saved per 100,000 population

These two countries, however, have different incidence and prevalence rates of HIV. These epidemiologic factors, as well as other drivers of regional variation in HIV burden and treatment, likely explain the different estimates in lives saved per 100,000 population per unit increase in ART coverage. However, the estimate of 5.11 lives saved per 100,000 population can represent a starting point in estimating the health impact of increasing ART coverage in the LMIC context.

In a context of low HIV burden, the literature reports a lower number of lives saved per 100,000 population relative to the high HIV burden context. Specifically, using Latin American countries classified as middle income, one report finds that for every 1 percentage point increase in ART coverage, there are 0.3 fewer AIDS-related deaths per 100,000 population [22]. This estimate is also used in the lives saved calculator for a “low HIV burden” context option.

B. Lives saved due to ART to prevent mother-to-child transmission

WHO estimates that in the absence of ART, ~30% of mothers transmit HIV to their infants (with a range of 15% to 45%) [23]. With ART for PMTCT, this transmission rate can be reduced to below 5%. In 2014, 21 priority sub-Saharan African countries provided ART to 77% of pregnant women living with HIV [24]. Among these women, mother-to-child HIV transmission fell to 9%.

Using the estimates above, in the absence of ART for PMTCT, for every 100 HIV-positive pregnant women, ~30 cases of HIV-positive infants are estimated. An estimated 50% of children living with HIV die before their second birthday. In the absence of ART for PMTCT, for every 100 HIV-positive pregnant women, there would be 15 child deaths (i.e., 50% $*$ 30 cases). With ART for PMTCT, for every 100 HIV-positive pregnant women, ~5 children would be HIV-positive, of which 50% (i.e., 2.5) are estimated to die prematurely.

Based on these calculations, the number of HIV-related child deaths averted for every 100 additional HIV-positive women on ART for PMTCT would be 12.5 (i.e., 15 child deaths without ART for PMTCT minus 2.5 child deaths in the presence of ART for PMTCT). The model can input country-specific ART for PMTCT coverage levels to estimate child lives saved per percentage point increase in ART for PMTCT coverage.

C. Lives saved due to TB treatment coverage

A report by the Global Fund [25] includes the following assumption for lives saved due to TB treatment coverage:

Currently it is assumed that one life is saved for every three cases of TB that are treated, based on the difference in the case fatality ratio of treated and untreated TB.

The 3:1 ratio could be applied to country-level estimates of total TB cases that are treated. For example, Sri Lanka shows 64% TB treatment coverage and ~10,000 detected cases per year [26]. Using these inputs, the total estimate of all TB cases is 15,625 (i.e., 10,000 / 0.64). Sri Lanka's stated TB treatment success rate is 85%. Given these inputs, the current TB treatment coverage (i.e., 10,000 identified cases ∗ 0.85) leads to 8,500 successfully treated cases. If we increase TB treatment coverage by 1 percentage point, this increase will show 8,633 successfully treated cases – or an additional 133 (i.e., 8,633 – 8,500) cases treated. Application of the 3:1 ratio to these 133 newly treated cases yields 44.3 lives saved per year in Sri Lanka associated with a 1 percentage point increase in TB treatment coverage.

D. Lives saved due to seeking cure for fever among children under 5

The WHO's World Malaria Report [27] found that in sub-Saharan Africa, 36% of febrile children are not brought to treatment facilities for care. Based on this statistic, 64% of children (i.e., 100% minus 64%) sought treatment for a fever at any facility. Moreover, the proportion of febrile children who received a malaria diagnostic test in the public sector rose from 29% in 2010 to 51% in 2015, yielding a percentage increase of (51 – 29) / 29 = 76%.

If a child seeks treatment for fever at a facility in LMICs, it is assumed that the child would receive a diagnostic test. Over the same time period, under-5 mortality due to malaria declined by 35%. A decline in malaria is assumed to be related to treatment seeking for fever. Given these parameters, every 1 percentage point increase in children under 5 years with fever seeking treatment at any facility was assumed to correspond with a 0.46% decline (i.e., 35 / 76) in malaria mortality for under-5 children. The model can factor in estimates of malaria mortality for under-5 children in 1 year and multiply them by 0.0046 (i.e., 0.46%) to estimate children's lives saved for each 1 percentage point increase in children seeking care for fever.

E. General considerations on inputs needed and assumptions of the model

Empirical literature was used to derive estimates of lives saved due to increased HIV, TB, and malaria treatment service coverage. The gains in service coverage for four specific treatments are positively associated with DNM concentration, which supports the notion that investments in DNMs result in additional lives saved. The lives saved calculations, albeit omitting more granular information on the health services landscape and the dynamics of HIV, TB, and malaria, rely on a set of relatively straightforward epidemiological assumptions. For each indicator, Table 2 in the main manuscript illustrates the additional lives saved by a 1 percentage point increase in coverage, the input(s) needed by the model to estimate lives saved and the key assumptions for that estimate.

As the table shows, the number and level of complexity of the inputs required by the model is modest. We remind the reader of the important caveat that correlation estimates do not represent causal relationships. Rather, the model illustrates the potential health impact that may be realized from additional HRH investments. Including the lower and upper bound estimates of lives saved further underscores the uncertainty inherent in these estimates.

Part 2: Targeted literature review to identify empirical estimates for specific assumptions in the model

A. CHW productivity

While estimates of the empirical relationship between health worker density and service coverage for HIV/AIDS, TB and malaria were based on actual cross-country data on density for DNMs, cross-country data for CHWs were less complete and insufficient for separately estimating an empirical relationship with service coverage. This is a notable limitation of the available data to date, and an area where this work can benefit from refinement in the future as more data on additional occupational groups become systematically available.

To be able to estimate how HRH investments in CHWs affect health outcomes, a productivity multiplier was used that reflects the ratio of the productivity of a doctor or nurse/midwife to the combined productivity of CHWs and a doctor or nurse/midwife providing collaborative care. A literature search was conducted to identify studies that provided suitable estimates of this relative productivity. The literature review searches were performed using the WHO and International Labour Organization's definition of a CHW:

Community health workers provide health education and referrals for a wide range of services, and provide support and assistance to communities, families and individuals with preventive health measures and gaining access to appropriate curative health and social services. They create a bridge between providers of health, social and community services and communities that may have difficulty in accessing these services [28].

Given the definition, the terms "community healthcare worker", "community health worker", "lay health worker", "community-based health worker" and "task shifting" were searched. Search results indicated that there is an expansive literature examining the efficacy of CHWs in providing healthcare. To identify estimates that would allow us to convert the productivity between DNMs and the collaborative care provided by nurses, midwives and CHWs, the literature review focused on quantitative studies that examine task sharing. Many of the studies in the task-sharing literature involved simulation and estimation of DNM and CHW stock [29], which are not applicable for this project. However, two relatively recent systematic literature

reviews were identified on studies that conducted CEA on CHW involvement and task sharing [13,14]. The studies included in these two systematic literature reviews were evaluated for reliable estimates of the effectiveness of CHW, because CEA studies require specific estimates on effectiveness of the intervention in question and the comparator. Estimators identified specifically measured sharing of tasks between doctors, nurses and midwives, or DNMs and CHWs (as opposed to DNMs shifting the tasks in their entirety to CHWs). The focus on these estimates enabled comparison of the changes in effectiveness of DNMs and CHWs providing collaborative care, compared to only DNMs providing care. The changes in effectiveness between the two groups were then translated into changes in overall productivity in each task sharing intervention, thus enabling direct comparison of relative output among the groups.

Of the CEA studies reviewed, those with the following criteria were included:

- The intervention focused on task sharing between CHWs and professionally trained healthcare workers (i.e., DNMs).
- CHW involvement was the only part of the intervention. In other words, no other interventions were examined simultaneously.
- Outcome variables could be translated to productivity, focusing on service coverage (e.g., TB cure rate, capacity of TB treatment programme).
- All the estimates provided and used in the CEA resulted from primary data collected by the study, rather than from secondary data analysis (e.g., in simulations) on previously published estimates. In one of the studies included in the systematic literature review [30], the primary data was reported in a separate study. Therefore, that study was reviewed to extract the estimates, which were then translated to productivity for this analysis.

- The estimates represent statistically significant differences in service coverage between the task-sharing group and the MDs, NMs or DNM only groups.

Consequently, the exclusion criteria included the following:

- studies that only examined changes in prevalence and did not report service coverage outcomes;
- studies that had costs but no estimates of effectiveness; and
- studies that used estimates generated from other studies.

If the target of HRH investments is CHWs and users enter the number of CHWs to be targeted, the number of CHWs is then converted to their DNM equivalent (unobserved to the user). The subsequent calculations then proceed according to the estimates used for DNMs. Table 5 below presents the study estimates, converted productivity multipliers and the final median productivity multiplier included in the Excel calculator.

Table 5. Studies that quantitatively estimate the relative productivity of DNMs to CHWs

Citation	Outcome measure	Performance outputs		Multiplier
		DNMs	CHWs	(DNMs/CHWs)
Buttorff et al. [31]	Reduction in psychiatric symptoms	39.9%	67.5%	1.02
Dick [12]	Treatment completion	64.3%	83.0%	1.187
Jafar et al. [11]	Medication use	46.3%	49.7%	1.034

Sabin et al. [32]	Death averted	–	17.9 / 1000	1.179
**Median**				**1.1065**

CHWs: community health workers; DNMs: doctors, nurses and midwives

B. Studies on pre-service education

There is an absence of studies which examine the relationship between the number of medical professional school enrollees and the number of graduates in LMICs. Among quantitative studies assessing interventions that increased the number of enrollees and graduates in medical education, none were found that generated a usable number for this project. The literature on pre-service education contains mostly qualitative and prescriptive studies outlining the need to train and retain HRH in low-income settings. One study examined Ethiopia's "flood and retain" policy for increasing the supply of HRH by building professional schools [30]. The qualitative interviews conducted in the study show that the government had increased the number of students, but not the number of teachers, equipment and other resources. In general, these studies highlight the need to ensure that there are enough resources to recruit and retain HRH; simply increasing pre-service education is insufficient [33]. These findings underscore the importance of including provisions for eventual full health worker salary support for any HRH investment in pre-service education that aims to augment the inflows of workers into the HRH labour supply.

In addition, these studies provide no relational estimates between enrolment, graduation and DNM density, most likely due to the lack of transparency and systematic data collection [34]. A study surveyed all identified medical schools in sub-Saharan Africa and found that 81% of responding schools indicated that they had no tracking system for their graduates and could not determine whether they were practicing medicine and where [35]. Consequently, there is a lack

of data to determine the relationship between healthcare-related professional school enrolment and its resulting effect on labour force inflows.

One study conducted by WHO collected data on the number of new graduates in 12 countries in Africa [36]. This study provides the most comprehensive information on the number of qualified HRH personnel in sub-Saharan Africa; but the information is somewhat outdated and still not comprehensive enough for use in this exercise.

C. Studies on salary pay raises and retention

There is an extensive literature on the labour market and the impact of wages on labour supply. Nevertheless, most studies that apply labour market theories on HRH in LMICs are theoretical [2,3] and thus do not provide usable empirical estimates for this project. Furthermore, studies which calculated specific elasticity estimates for healthcare workers in LMICs are scarce. In terms of quantitative analysis, only a few studies [37] have generated estimates on the relationship between remuneration and training and retention from regression models. As is evident in findings from numerous systematic literature reviews [38,39,40] studies examining the relationship between remuneration and training and retention and performance suffer from several limitations:

- Many studies are purely descriptive, that is, survey- and questionnaire-based studies which highlight the importance of remuneration and training, but provide no estimates on the relationship between variables [41].
- The primary outcome of focus is often the satisfaction of healthcare workers with their career and position [42]. These outcomes are not connected to retention or performance and cannot be translated into elasticity estimates.

A broad literature search and review showed the type of quantitative analysis most useful in the context of this project to be findings from DCEs in LMICs. For this reason, our subsequent literature search focused on DCE studies. The following terms were searched individually and in combination: "remuneration", "wages", "healthcare work force", "human resources for health", "doctors", "nurses", "supply-oriented interventions", "DCE", "training", "development", "retention" and "low- and middle- income". Posters and PowerPoint presentations were excluded, as studies presented in these formats do not provide adequate information for us to determine the usability and reliability of the estimates generated.

Published studies of the effect of HRH investments on HRH density were examined to identify estimates translatable to several outcomes: (i) retention in the country, (ii) willingness to work in poorly equipped positions, (iii) remaining at a specific location (e.g., rural) or position and (iv) relocation to a rural/remote location. Retention in the healthcare sector or the country are the most relevant outcomes for extracting an estimate of the effect of salary pay raises on HRH availability via retention among currently trained HCWs. In qualitative interviews conducted to outline attributes for DCEs, the outcomes for willingness to work in poorly equipped positions has consistently emerged as a salient characteristic influencing individual decisions to leave or remain in a country.

Theoretically, these various outcomes can be translated into an equivalent number of FTEs, which can then be used to calculate HRH density (further explanation follows). To ensure the relevance of the findings and their applicability to the current HRH context, estimates in our final model were restricted to studies published between 2008 and 2019.

Within the included studies, estimates of the relationship between remuneration and training and retention generally surveyed current healthcare workers or students in medical or nursing schools in LMICs. Many of the DCE studies also calculated the willingness-to-pay (WTP) value that healthcare workers were willing to trade for other job characteristics. The WTP

measurement is useful, as it provides a monetary value for how much a person is willing to pay for a given good or experience or to avoid an undesired outcome. In the context of HRH, the WTP value provides a monetary value of how much it is worth for a healthcare worker to stay or leave his/her current position, thus affecting the DNM labour supply. The WTP value is calculated as the percentage of the average salary of each occupational group, which indicates the additional resources needed to retain a healthcare worker.

This body of literature is the most applicable for extracting estimates on how increases in remuneration and training may lead to increased HRH density. The included estimates are presented in Table 6 below.

Table 6. Studies examining the association between salary pay raises and willingness to work in poorly equipped facilities

**Country category**	**Doctors**	**Nurses and midwives**
Low	Three studies:<br>• *Ethiopia: 27% increase in base salary leads to selection of remote post.*<br>• Ethiopia: 26% increase in base salary leads to staying in condition with poorer equipment.<br>• *Malawi: Two times current monthly salary leads to*	Six studies:<br>• *Ethiopia: 72.2% increase in base salary leads to selection of remote post.*<br>• Ethiopia: 50% increase in base salary leads to staying in conditions with poorer equipment.<br>• *Malawi: MWK 10 000 (US$ 10) increase in salary leads to*

	selection of work in rural areas.<br>• Uganda: Additional USh 1 million (US$ 426) (1.43 times the base salary) per month leads to a 46% increase in preference for poorer-quality facilities.	*increase in choosing rural post by 50% point change.*<br>• *Tanzania: 80–100% increase in salary leads to selection of rural post.*<br>• *Tanzania: TZS 57 151 (US$ 25) leads to selection of remote post.*<br>• Uganda: Additional USh 1 million (US$ 426) per month leads to an increase in 17% in preference for poorer-quality facilities.
Lower middle	Three studies:<br>• *India: 33% increase in salary increases the likelihood of doctors accepting a rural post by 13% point change.*<br>• *Kenya: 20% increase in salary increases the likelihood of doctors accepting a rural post by 22.8% point change.*<br>• *Viet Nam: Doctors are willing to pay 7.04 million VND*	Three studies:<br>• *India: INR 10 000 (US$ 140) increase in salary increases the likelihood of nurses accepting a rural post by 31% point change.*<br>• *India: 2.5 times current salary leads to 61% point change in acceptance of rural positions.*<br>• *Indonesia: IDR 7 million (US$ 503) increase in salary leads to selection of remote post.*

	(US$ 303) to be located in an urban post.	
Upper middle	Five studies:<br>• China: 10.8% of current income leads to increased likelihood of doctors choosing to work in less well-equipped health centres.<br>• *China: Doctors are willing to pay CNY 4020 (US$ 496) to be located in an urban post.*<br>• Iran: 36.5% salary increase (or additional US$ 730) leads doctors to be willing to work in poorer facilities.<br>• *Peru: PEN 1000 (US$ 300) increase/month increases the odds ratio of staying in a rural post by 2.82.*<br>• *Thailand: 45% increase in salary leads to selection of rural post.*	Four studies:<br>• China: 8.1% of current income leads to increase in the likelihood of nurses choosing to work in less well-equipped health centres.<br>• *China: CNY 3000 (US$ 444) increase in income leads to 75.2% of nurses choosing a rural job.*<br>• *Peru: PEN 1000 (US$ 300) increase/month increases the odds ratio of staying in a rural post by 2.95.*<br>• *South Africa: ZAR 250 000 (US$ 18 475) annual salary leads to choosing to remain at current position.*

Studies in italics are excluded as they do not meet inclusion criteria.

There were three studies (from LMICs that included doctor and/or nurse/midwife occupational groups) that met the inclusion criteria (see Table 7 below for further details) [4,5,6]. However, only one of the three studies actually reported the fraction of study participants willing to accept and stay for the pay raise offered – data required to actually calculate an estimate and make the study usable.

Table 7. Included studies on salary pay raise and retention

**Study**	**% salary increase**	**% of respondents willing to accept**	**Estimator**
Hanson and William [4]	26% for D<br>50% for NMW	–	
Rockers et al. [5]	71% for NMW	46%	1.54
Song et al. [6]	11% for D<br>8% for NMW	–	

Rockers et al. [5] reported that given a 71% increase in pay, 46% of medical students would be willing to work in a lower-quality facility. Dividing the increase in pay that would incentivize working in a poorer-quality location by the percentage found to accept the incentive converts this association to FTEs: 71% / 46% = 1.54. This quotient is interpreted as a 54% increase in salary being necessary to retain 1 FTE. Given the limited number of studies, the estimates across income categories and across occupational groups relied on one overall estimate (1.54), which can be applied to all groups.

D. Studies on in-service training and retention

A literature search was conducted to identify DCE studies that include in-service training and outcomes related to retention. Given the few studies that focus on in-service training, the content, duration, cost or frequency of in-service training is not considered further.[3] A review of the available empirical estimates showed that there are few to no studies that report any results relating in-service training to worker retention. Thus, the empirical basis for this investment pathway is not supported by the current state of the literature.

---

[3] Training can vary substantially across settings, health conditions and occupational groups. However, most studies do not provide enough information to characterize the content and intensity of training in order to make more specific estimates of these attributes.

Assumptions of the "lives saved" calculator

The calculator includes options for specifying HRH investments for four main investment types:

- pre-service education aimed at increasing the number of health workers entering the health-care labour market from medical training programmes;
- salary pay raises, broadly defined as any change in base compensation (wages, benefits) given to existing health workers;
- incentives (e.g., pay for performance) provided for meeting target performance outputs to increase the productivity of individuals privy to these schemes; and
- in-service training for existing health workers to increase the skill and quality of services a given health worker can produce.

Conceptually, pre-service education and salary pay raises are thought to affect the HRH labour market on the extensive margin, or the availability of HRH workers to be employed in a health service delivery capacity. This includes increasing the number of health workers who may enter the labour market either by retaining (through salary pay raises) health workers in service delivery positions who would otherwise have exited, or by encouraging employed workers to put in more hours for those who may be sub-optimally employed (and thus could be measured in terms of fractions of FTEs).

Incentives and in-service training investments can affect the HRH labour market on the intensive margin, or the productivity (services delivered per worker) of labour. While theoretically in-service training (viewed as an occupational benefit) may also influence health workers' decision to join the health workforce labour market or increase their hours worked on the extensive margin, our search of the literature found no empirical data that could be used to estimate this relationship.

1. Investments are made on top of existing HRH workforce dynamics. It was assumed that the labour market operates at a steady state in which inflows (newly graduated workers or immigrants) and exits (due to death, departure or retirement) occur as they historically have occurred.

2. The labour market in LMICs is currently operating at full capacity. Many countries in LMICs do not have enough demand to support a larger HRH workforce [43]. As such, any additional workers to be employed will need to be fully supported for compensation, whether for new graduates from medical education (above and beyond steady-state inflows) or for retaining workers in their current position.

3. The user can input the number of workers targeted for the investment. To calculate the association between HRH investments and treatment service coverage, the starting point for calculating the impact of investments will be the number of health workers targeted by occupational group and, for the case of pre-service education and salary pay raises, the relative size of investment to be made (i.e., 10% increase in salary, increasing the number of graduates to be supported in the labour market by 20%).

4. The calculator will not estimate the cost implications of the investments. The costs of HRH investments will vary considerably across country and health system contexts. In addition, it is assumed that the salary support given to health workers is discounted over the full lifetime of each worker.

5. Investment effects will be static. While all investments will take time to implement and to take effect in the health system, the estimates are simplified to reflect the result after all effects have worked through the system.

6. The main effects of different HRH investment options will be additive. If multiple investment types are chosen, resulting estimates of FTEs will be assumed to be linearly additive in a steady-state labour market.

7. Estimates will be stratified by occupational group (DNMs and CHWs) and country income level (low, lower-middle and upper-middle) to the extent supported by the empirical literature. Because associations of HRH investments with density and performance may vary substantially by occupational group and the depth of the labour market for health workers across income levels, calculations will be sensitive to occupational group targeted by country income level to the extent possible. Note that given the data requirements of using all countries with available data (including those beyond LMICs) to estimate the relation between HRH density and service coverage levels, identified associations reflect an average across all country income levels.

8. Estimates for CHWs will be based on a productivity multiplier. Data on the availability of CHWs across countries is not as readily available as it is for DNMs. The peer-reviewed literature will therefore be used to estimate CHW performance using a productivity multiplier (see earlier section). For countries without CHW stock data, the income-level strata median ratio of DNM density to CHW density will be used, calculated from the set of countries for which data for all three health worker categories are available.

9. Estimates for clinical officers (COs), a default option for an additional category of healthcare workers, will be based on a productivity multiplier. This productivity ratio is estimated from a study which calculates sub-Saharan Africa CO salaries relative to nurse salaries (i.e., US$ 1915 / US$ 1865 = 1.026). [44] By default, the productivity ratio of COs relative to nurses and midwives is 1.03. The number of COs will be multiplied by the productivity ratio and then folded into the total number for nurses and midwives. The lives saved from additional COs will therefore be calculated in terms of nurses and midwives.

10. Associations between investment options and HRH availability or productivity will be based on aggregated estimates from the empirical literature. Estimates from different empirical sources are standardized for use in the calculator.

11. HRH availability and productivity are the intermediate outputs that feed into service coverage outcomes and, ultimately, into estimates of impact in terms of lives saved. Based on a review of global HRH data, past experience and a literature search on empirical estimates of association elasticities to be estimated by the calculator, estimates for HRH availability and productivity measures can plausibly be calculated across country contexts. Although other measures of HRH distribution have been developed, they have not been consistently calculated and used across settings and require significantly more data (e.g., comparable subnational units) in order to compute. Thus, HRH distribution measures are excluded from the set of HRH investment outcomes.

12. Service coverage increases resulting from HRH productivity investments will not be occupation specific. The optimal skills mix for producing health services, whether specific to HIV, TB, malaria or more generally, is unknown and likely to be highly specific to health systems structures in different countries. Additional assumptions would need to be made to identify the relative attribution of service coverage increases due to HRH productivity across health workforce categories. Furthermore, no assumptions or limits are made about scale economies, the marginal rates of substitution between capital and labour inputs or the pathway for labour market expansion in the production function for health.

13. There are diminishing returns to HRH availability investments on service coverage outcomes. This relationship is reflected in the log functional form transformation used in the regression analysis for Step 2 (see earlier section of this supplementary file). The assumption of

diminishing returns is not applied to service coverage increases resulting from HRH productivity investments, as there is no empirical basis for this from the existing literature.

14. The empirical relationship between HRH density and service coverage is based on regression results that aggregate DNMs. While regression estimates were analysed for doctors and nurses/midwives separately, the resulting statistical significance of these relationships did not hold as strongly across service coverage indicators as analyses that combined these groups.

15. The relationship between HRH inputs and HRH availability and productivity are measured without uncertainty estimates. Given that a key goal of the calculator involves arriving at a point estimate of lives saved due to HRH investments, we used the peer-reviewed literature as well as regression-based parameter estimates to derive the quantitative inputs. Whereas we acknowledge that each of these inputs is measured with error, for ease of interpretation of the calculator tool we did not apply formal uncertainty analyses (e.g., the delta method) to the standard errors of each input. Rather, we used only the standard error estimates from the regressions in Table 3 above) to arrive at lower and upper bounds of total lives saved in the calculator tool.

References

1. WHO. Lives saved calculator. WHO/UHL/HWF/HWP/2022.1. Available from https://apps.who.int/iris/bitstream/handle/10665/351524/WHO-UHL-HWF-HWP-2022.1-eng.xlsm

2. Andalón M, Fields G. A labor market approach to the crisis of health care professionals in Africa. IZA discussion paper no. 5483. 2011 (https:// papers.ssrn.com/sol3/papers.cfm?abstract_id=1765648, accessed 19 July 2021).

3. Scheffler R, Bruckner T, Spetz J. The labour market for human resources for health in low-and middle-income countries. Human Resources for Health Observer no. 11. Geneva: World Health Organization; 2012.

4. Hanson K, William J. Incentives could induce Ethiopian doctors and nurses to work in rural settings. Health Affairs. 2010;29:1452–60.

5. Rockers PC, Jaskiewicz W, Wurts L, Kruk ME, Mgomella GS, Ntalazi F et al. Preferences for working in rural clinics among trainee health professionals in Uganda: a discrete choice experiment. BMC Health Serv Res. 2012;12:212.

6. Song K, Scott A, Sivey P, Meng Q. Improving Chinese primary care providers' recruitment and retention: a discrete choice experiment. Health Policy Plan. 2013;30:68–77.

7. Rowe AK, Rowe SY, Peters DH, Holloway KA, Chalker J, Ross-Degnan D. Effectiveness of strategies to improve health-care provider practices in low-income and middle-income countries: a systematic review. Lancet Glob Health. 2018;6(11):e1163–75.

8. Perez-Cuevas R, Guiscafre H, Munoz O, Reyes H, Tome P, Libreros V et al. Improving physician prescribing patterns to treat rhinopharyngitis: intervention strategies in two health systems of Mexico. Soc Sci Med. 1996;42(8):1185–94.

9. Rowe SY, Peters DH, Holloway KA, Chalker J, Ross-Degnan D and Rowe AK. A systematic review of the effectiveness of strategies to improve health care provider performance in low- and middle-income countries: methods and descriptive results. PLoS ONE. 2019;14:e0217617.

10. Health Care Provider Performance Review [website]; 2021 (https://www.hcpperformancereview.org/, accessed 1 March 2021).

11. Jafar TH, Islam M, Bux R, Poulter N, Hatcher J, Chaturvedi N. Cost-effectiveness of community-based strategies for blood pressure control in a low-income developing country: findings from a cluster-randomized, factorial-controlled trial. Circulation. 2011;124:1615–25.

12. Dick J, Clarke M, van Zyl H, Daniels K. Primary health care nurses implement and evaluate a community outreach approach to health care in the South African agricultural sector. Int Nurs Rev. 2007;54(4):383–90. doi:10.1111/j.1466-7657.2007.00566.x.

13. Seidman G, Atun R. Does task shifting yield cost savings and improve efficiency for health systems? A systematic review of evidence from low-income and middle-income countries. Hum Res Health. 2017;15(1):29.

14. Vaughan K, Kok MC, Witter S, Dieleman M. Costs and cost-effectiveness of community health workers: evidence from a literature review. Hum Res Health. 2015;13(1):71.

15. Okello D, Floyd K, Adatu F, Odeke R, Gargioni G. Cost and cost–effectiveness of community-based care for tuberculosis patients in rural Uganda. Int J Tuberc Lung Dis. 2003;7:S72–9.

16. Marais BJ, Lönnroth K, Lawn SD, Migliori GB, Mwaba P, Glaziou P. Tuberculosis comorbidity with communicable and non-communicable diseases: integrating health services and control efforts. Lancet Infect Dis. 2013;13(5):436–48.

17. Duarte R, Lönnroth K, Carvalho C, Lima F, Carvalho ACC, Munoz-Torrico M. Tuberculosis, social determinants and co-morbidities (including HIV). Pulmonology. 2018;24(2):115–9.

18. WHO policy on collaborative TB/HIV activities: guidelines for national programmes and other stakeholders. Geneva: World Health Organization; 2012.

19. Wood R, Middelkoop K, Myer L, Grant AD, Whitelaw A, Lawn SD. Undiagnosed tuberculosis in a community with high HIV prevalence: implications for tuberculosis control. Am J Respir Crit Care Med. 2007;175(1):87–93.

20. Wang H, Wolock TM, Carter A, Nguyen G, Kyu HH, Gakidou E. Estimates of global, regional, and national incidence, prevalence, and mortality of HIV, 1980–2015: the Global Burden of Disease Study 2015. Lancet HIV. 2016;3(8):e361–87.

21. Granich R, Gupta S, Hersh B, Williams B, Montaner J, Young B et al. Trends in AIDS deaths, new infections and ART coverage in the top 30 countries with the highest AIDS mortality burden; 1990–2013. PLoS ONE. 2015:10(7);e0131353.

22. Gonzalez MA, Martin L, Munoz S, Jacobson JO. Patterns, trends and sex differences in HIV/AIDS reported mortality in Latin American countries: 1996–2007. BMC Pub Health, 2011;11(1):605.

23. Mother-to-child transmission of HIV. In: WHO/Teams/Global HIV Programme [website]. Geneva: World Health Organization; 2021 (https://www. who.int/teams/global-hiv-hepatitis-and-stis-programmes/hiv/prevention/mother-to-child-transmission-of-hiv, accessed 13 November 2021).

24. 2015 progress report on the Global Plan towards the elimination of new HIV infections among children and keeping their mothers alive. Geneva: UNAIDS; 2015

(http://www.unaids.org/sites/default/files/media_asset/JC2774_2015ProgressReport_GlobalPlan_en.pdf, accessed 19 July 2021).

25. Report of the first meeting of the expert panel on health impact of Global Fund investments. Geneva: The Global Fund; 2014 (https://www.theglobalfund.org/media/8049/corporate_expertpanelhealthimpactinvestmentsmeeting_report_en.pdf, accessed 19 July 2021).

26. Dissanayake C. Sri Lanka fast “developing” into TB hub of Asia. Sunday Times, 30 March 2014 (http://www.sundaytimes.lk/140330/news/sri-lanka-fast-developing-into-tb-hub-of-asia-91034.html, accessed 19 July 2021).

27. World malaria report 2016. Geneva: World Health Organization; 2016 (https://apps.who.int/iris/bitstream/hand le/10665/252038/9789241511711-eng.pdf, accessed 1 August 2021).

28. WHO guideline on health policy and system support to optimize community health worker programs. Geneva: World Health Organization; 2018 (https://apps.who.int/iris/bitstream/handle/10665/275474/9789241550369-eng.pdf?ua=1, accessed 22 February 2019).

29. Fulton BD, Scheffler RM, Sparkes SP, Auh EY, Vujicic M, Soucat A. Health workforce skill mix and task shifting in low income countries: a review of recent evidence. Hum Resour Health. 2011;9(1):1.

30. Mengistu BS, Vins H, Kelly CM, McGee DR, Spicer JO, Derbew M. Student and faculty perceptions on the rapid scale-up of medical students in Ethiopia. BMC Med Educ. 2017;17(1):11.

31. Buttorff C, Hock RS, Weiss HA, Naik S, Araya R, Kirkwood BR. Economic evaluation of a task-shifting intervention for common mental disorders in India. Bull World Health Organ. 2012;90:813–21.

32. Sabin LL, Knapp AB, MacLeod WB, Phiri-Mazala G, Kasimba J, Hamer DH. Costs and cost–effectiveness of training traditional birth attendants to reduce neonatal mortality in the Lufwanyama Neonatal Survival study (LUNESP). PLoS ONE. 2012;7(4):e35560.

33. Willcox ML, Peersman W, Daou P, Diakité C, Bajunirwe F, Mubangizi V. Human resources for primary health care in sub-Saharan Africa: progress or stagnation? Hum Resour Health. 2015;13(1):76.

34. Pemba S, Macfarlane SB, Mpembeni R, Goodell AJ, Kaaya EE. Tracking university graduates in the workforce: information to improve education and health systems in Tanzania. J Pub Health Policy. 2012;33(1):S202–25.

35. Mullan F, Frehywot S, Omaswa F, Buch E, Chen C, Greysen SR. Medical schools in sub-Saharan Africa. Lancet. 2011;377(9771):1113–21.

36. Kinfu Y, Dal Poz MR, Mercer H, Evans DB. The health worker shortage in Africa: are enough physicians and nurses being trained? Bull World Health Organ. 2009;87(3):225–30.

37. Kolstad JR. How to make rural jobs more attractive to health workers. Findings from a discrete choice experiment in Tanzania. Health Econ. 2011;20(2):196–211.

38. Dambisya YM. A review of non-financial incentives for health worker retention in east and southern Africa. EQUINET discussion paper no. 44. 2007:49–50.

39. Willis-Shattuck M, Bidwell P, Thomas S, Wyness L, Blaauw D, Ditlopo P. Motivation and retention of health workers in developing countries: a systematic review. BMC Health Serv Res. 2008;8(1):247.

40. Henderson LN, Tulloch J. Incentives for retaining and motivating health workers in Pacific and Asian countries. Hum Resour Health. 2008;6(1):18.

41. Kotzee TJ, Couper ID. What interventions do South African qualified doctors think will retain them in rural hospitals of the Limpopo province of South Africa? Rural Remote Health. 2006;6(3):581.

42. Agyepong IA, Anafi P, Asiamah E, Ansah EK, Ashon DA, Narh-Dometey C. Health worker (internal customer) satisfaction and motivation in the public sector in Ghana. Int J Health Plann Manage. 2004;19(4):319–36.

43. Liu J, Goryakin Y, Maeda A, Bruckner T, Scheffler R. Global health workforce labor market projects for 2030. Hum Resour Health. 2017;15(1):11. https://doi.org/10.1186/s12960-017-0187-2.

44. McCoy D, Bennett S, Witter S, Pond B, Baker B, Gow J. Salaries and incomes of health workers in sub-Saharan Africa. Lancet. 2008;371(9613):675–68.
