# Supplementary material for: A novel approach to estimate the impact of health workforce investments on health outcomes through increased coverage of HIV, TB and malaria services
Source: Hum Resour Health. 2023 Aug 21;21:67. doi: 10.1186/s12960-023-00854-0 (PMC10441693; doi:10.1186/s12960-023-00854-0)
Supplement: Supplementary file 2 — Additional file 2. Approaches to estimating the relationship between human resources for health (HRH) density and service coverage. [file 12960_2023_854_MOESM2_ESM.pdf]

## Approaches to estimating the relationship between human resources for health (HRH) density and service coverage

### A. Data envelopment analysis (DEA) for the benchmarking alternative

Data envelopment analysis is a nonparametric estimation strategy that can be used for identifying a country's efficiency in covering treatment services at particular levels [1]. DEA borrows from tools in economics and operations management to identify groups that maximize the utility of their existing resources to achieve a desired end. In our scenario, countries serve as the "group" and DNMs per 1000 are the "inputs" that produce treatment service coverage for HIV, TB and malaria. Countries are then ranked by DEA according to an efficiency score, which is calculated by the successful attainment of a certain threshold of treatment coverage per DNM worker in that country. DEA has been used by WHO in past reports to provide specific DNM benchmarks for attaining desired treatment coverage targets [2].

Performed using the "dea" command in STATA, DEA ranks all countries according to efficiency [3]. DEA was applied separately for each of the four treatment service coverage indicators. The 60% coverage level was chosen as a level of coverage at the medium range. The DEA then identified the top 20 countries as exemplary in their efficiency for attaining > 60% service coverage. The 20 countries included represented the Americas, Africa, and South-East Asia and the Western Pacific regions. If the mean value of DNM is taken from this list of 20 efficient countries (separately for each treatment coverage, given that countries may be efficient in, say, TB coverage but not in HIV coverage), the following benchmark levels of DNMs are produced at a treatment coverage level of 60%:

- ART coverage for HIV: 1.54 DNMs
- ART coverage for PMTCT: 0.64 DNMs

- TB treatment coverage: 0.77 DNM
- Children < 5 sought treatment for fever: 0.71 DNM.

Although these numbers are lower than previously published reports of desired DNMs, results cohere with the notion that DEA seeks efficiency and ranks countries favourably if they attain treatment service coverage with relatively fewer health workers.

With these DEA-derived DNM benchmarks at 60% treatment coverage, a log-linear function was fit through this point and the “cap” DNM level for 99% treatment coverage which was empirically identified previously (by taking the median DNM level of existing countries that attained > 90% treatment service coverage). The log-linear function implies a curvilinear relation between DNM and treatment service coverage, such that after a certain level of DNM investments, there are diminishing returns to increases in treatment service coverage. This cap was set to make it logically impossible for a country to achieve DNM levels higher than the level that the highest DNM countries in the present day have attained. In addition, it is assumed that no country could have a negative DNM (at low levels of treatment service coverage).

Results of this approach appear in the figures below (the x-axis is DNMs per 1000 population, and the y-axis is treatment coverage percentage). Note that the curves show diminishing returns at the higher end of treatment coverage, provide specific values of DNM across the entire distribution of treatment coverage and do not show any gross discontinuities (or jumps) in DNM as coverage percentage is increased.

If this logic is applied to the “Coverage Target” tab, for a country with fewer DNMs than indicated by these benchmark DNM levels for a particular treatment coverage percentage, the country’s desired value would move up to the benchmark DNM. Alternatively, if that country already has more DNMs than indicated by the benchmark, the “Coverage Target” tab does not suggest a higher level of DNMs than the country has already attained.

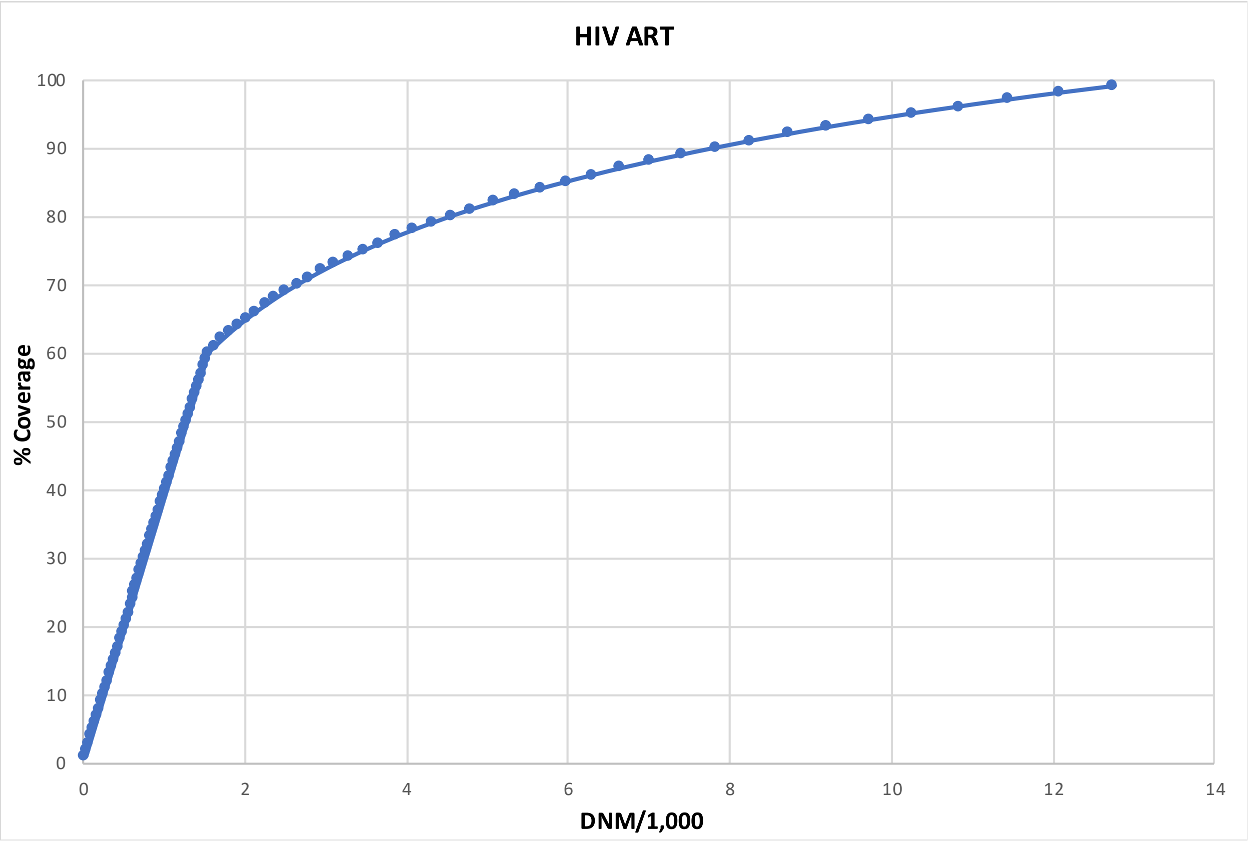


ART: antiretroviral therapy; DNMs: doctors, nurses and midwives

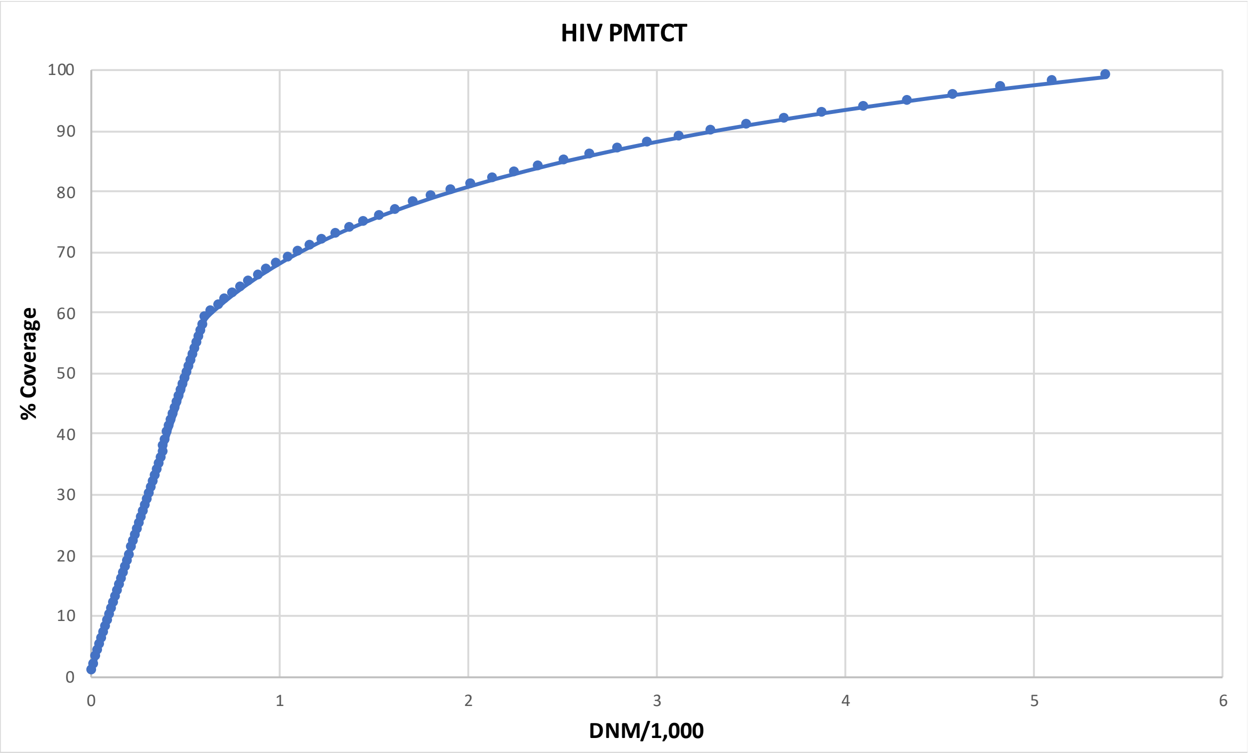


DNMs: doctors, nurses and midwives; PMTCT: prevention of mother-to-child transmission

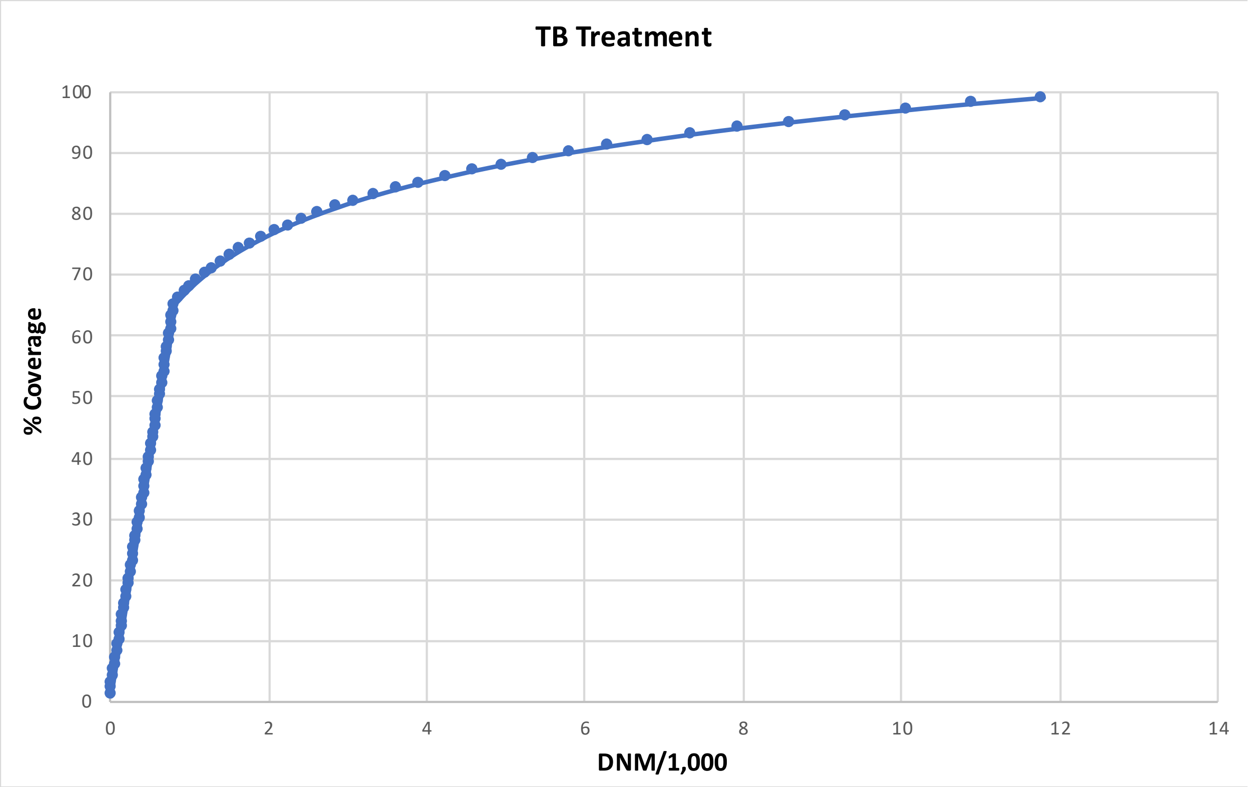

DNMs: doctors, nurses and midwives; TB: tuberculosis.

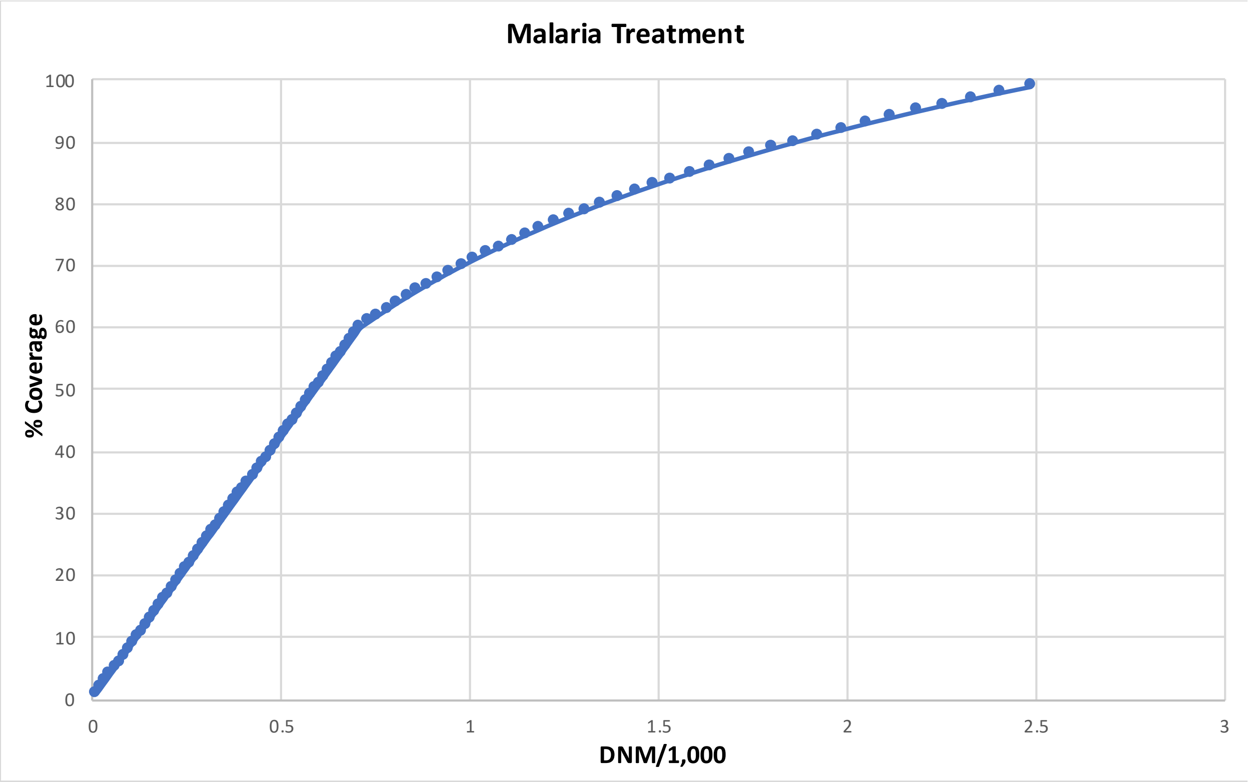


DNMs: doctors, nurses and midwives

## B. Aggregation of four treatment service indicators into a single metric

The "composite regression method" was explored as a method of integrating the four regression results from Table 1 in the main manuscript (i.e., one for each treatment service indicator shown to have an empirical association with DNM concentration) into a single metric. The benefit of the single metric is that one can produce a summary indicator of overall increase in treatment service coverage for HIV, TB and malaria associated with investment in DNMs. This approach has intuitive appeal as DNMs do not typically treat single conditions to the exclusion of other conditions. Thus, a single composite index may better capture the healthcare reality in which

treatment service coverage gains occur concurrently across HIV, TB and malaria as a result of augmenting DNM investments.

Four ways were explored for estimating the association between a composite treatment service coverage metric and DNM concentration.

1. Continuous index, unweighted

To create the composite index, the percentage attainment of the four treatment coverage indicators (Table 1 in the main manuscript) for each country was summed. For example, one country shows the following treatment coverage:

- ART coverage (% of people living with **HIV**): **64%**;
- Percentage of pregnant women with **HIV** who receive antiretroviral medicine for prevention of PMTCT: **80%**;
- **TB** treatment coverage: the number of new and relapse TB cases per number of incident cases: **45%**; and
- percentage of children < 5 years with fever who sought treatment at any facility (an indicator of treatment for **malaria**): **27.1%**.

The sum of these four percentages (i.e., 64 + 80 + 45 + 27.1) for this country produces a composite index score of 216.1.

This summation step was used for all countries. Next, this composite score was regressed as a function of log(DNM). In the basic analysis, the composite index is retained as a continuous measure. Results, shown in the "continuous, unweighted" row (Table 1 below), indicate a positive and statistically detectable ($P < 0.05$) association between DNM and this composite index.

**Table 1. Composite regression method results, four treatment indicators combined**

| Summary index | *N* | DNM | SE | *P* value | Intercept |
|---|---|---|---|---|---|

| | | coeff. | | | |
|---|---|---|---|---|---|
| Composite index (continuous, unweighted) | 62 | 21.60 | 7.57 | 0.006 | 226.02 |
| Composite index (DALY weight) | 62 | 20.52 | 7.86 | 0.01 | 216.97 |
| Composite index (median split, unweighted) | 62 | 0.50 | 0.17 | 0.005 | 1.79 |

2. Continuous index, DALY weighted

In LMICs, the burden of disease due to HIV exceeds that of either TB or malaria. Additional treatment service coverage for ART, therefore, could be considered as reducing more DALYs than would similar gains in treatment coverage for malaria due to children seeking care for fever. To adjust the continuous composite index results by the DALYs of the condition that each treatment service seeks to address, an additional DALY-weighting strategy is provided.

For LMICs only, specific rows were identified in the WHO Global Health Estimate (GHE) summary tables [4] for HIV, TB and malaria. Table 2 below lists the four treatment service coverage indicators, the specific row used in the Global Burden of Disease table and the total DALYs lost in LMICs due to that condition.

**Table 2. LMICs only: DALYs due to HIV, TB and malaria**

| Treatment coverage | GHE cause | Estimated DALYs ('000s) |
|---|---|---|
| • ART overall<br>• ART for PMTCT | HIV/AIDS | 59 139.70 |
| • TB treatment coverage | TB | 51 362.70 |
| • Children seeking treatment for fever | Malaria | 37 368.30 |

An "analytic weight" was calculated by dividing DALYs for a particular disease by the sum of DALYs from all three diseases. This process produces the following analytic weights:

**Table 3. Analytic DALY weight, by disease**

| Condition | Analytic DALY weight |
|---|---|
| HIV | 0.40 |
| TB | 0.35 |
| Malaria | 0.25 |

The DALY-weighted composite regression slightly adjusts results from the unweighted regression by "upweighting" the importance of attaining HIV treatment service coverage indicators, and by "downweighting" the malaria treatment service coverage indicator. Here, the TB DALY weight is the "base" weight. HIV treatment indicators are upweighted by a factor of 1.14 (i.e., 0.40 / 0.35), and malaria is downweighted by a factor of 0.71 (i.e., 0.25 / 0.35). Given the use of two HIV treatment indicators, each of these HIV indicators was weighted by 1.07. The TB service treatment indicator remained unchanged (i.e., 0.35 / 0.35, or a DALY weight of 1.0). After applying these analytic weights to each country's value for the treatment service coverage indicators, the regression equation was re-estimated. Results from the DALY-weighted analysis

appear very similar to the unweighted analysis (see Table 1 in the main manuscript), although the intercept and DNM coefficients are slightly smaller.

To assist the reader in contextualizing the composite index regression results from Table 1 above, Table 4 following next, provides a few fitted values of treatment service coverage for specific levels of DNMs. For example, 2.5 DNMs per 1000 population corresponds with an unweighted composite index of 246 (i.e., an average of 61.5% coverage per each of the four treatment service indicators, since $4 * 61.5 = 246$). In addition, raising the DNM level from 2.5 to 4.0 per 1000 population is associated with a 10 unit increase in the unweighted composite index.

**Table 4. Sample values of composite index coverage by level of DNMs**

| DNMs per 1,000 population | Composite index* (unweighted) | Composite index* (DALY weighted) |
|---|---|---|
| 1.0 | 226 | 217 |
| 1.5 | 234 | 225 |
| 2.0 | 241 | 231 |
| 2.5 | 246 | 236 |
| 4.0 | 256 | 245 |

* *Values rounded to the nearest integer.*

3. Median split

In the median split approach, each of the four continuous measures of treatment service indicators was converted into a binary variable: scores above the grand median for LMICs received a 1 and scores below the median received a zero. Next, the four binary indicator scores were summed to produce a composite index. The range of each country's median split

composite index is from zero to 4, with 4 indicating attainment of all four treatment indicators above the country medians.

The dependent variable in the OLS regression was specified as the median split composite index score, and the DNM coefficient was the independent variable. Results (last row of Table 1 above) show a positive association between DNM and the composite treatment service index. However, interpretation of the coefficient is challenging in that movement of the median split index from, say, 3 to 4 does not seem intuitive. Therefore, this analytical model was not retained in the development of the MS Excel calculator.

4. Expanded number of service coverage indicators

The last approach to producing a composite index assumes that increasing HRH investments may show "on the ground" improvements in treatment service coverage rates for HIV, TB and malaria in areas that do not show statistically detectable correlations in isolated OLS regressions using aggregate level data. In this scenario, three candidate treatment service indicators were added to the composite index. These candidate indicators focus on treatment modules for HIV, TB and malaria in LMICs which DNMs may directly provide to patients.

- percentage of HIV/TB coinfected population who receive ART;
- TB treatment success rate (% of new cases); and
- malaria: percentage of women aged 15–49 with a live birth who received 2+ doses of sulfadoxine-pyrimethamine (SP/Fansidar).

In total, for each country this process sums seven treatment service indicators: three new HIV, TB and malaria indicators, and the four "base" indicators that show empirically robust association with DNM concentration. Regression results from this augmented model are shown in Table 5 of this supplementary file. The *P* value indicates that the DNM coefficient for these seven treatment indicators does not reject the null; there is no association between DNM and the expanded composite index of treatment service indicators. In addition, only 38 countries

have non-missing values on all treatment service indicators. Imputation of median treatment service values for LMICs with missing treatment indicators produces regression results very similar to those shown in Table 5 with the larger country set.

**Table 5. Augmented composite regression results, seven treatment indicators**

| **Summary index** | ***N*** | **DNM coeff.** | **SE** | ***P* value** | **Intercept** |
|---|---|---|---|---|---|
| Composite index (continuous, unweighted) | 38 | 21.27 | 14.79 | 0.16 | 421.76 |

In addition, different transformations of the dependent and independent variables were assessed in the OLS regression. The inverse sine transformation for treatment coverage, previously implemented by WHO [5], was assessed, as well as a square root transformation for treatment coverage and DNM (one at a time). None of these transformations produces a DNM coefficient which rejects the null.

Based on this result, there is no empirically supported approach to expanding the list of treatment service indicators beyond the four indicators described in Table 1 in the main manuscript. Any inclusion of additional treatment service indicators for HIV, TB and malaria would require assumptions that WHO and the Global Fund, given their expertise in this area, are able to make about the relation between DNMs and service coverage.

## References

1. Brockett PL, Golany B. Using rank statistics for determining programmatic efficiency differences in data envelopment analysis. Manage Sci. 1966;42(3):466–72.

2. Health workforce requirements for universal health coverage and the Sustainable Development Goals. Human Resources for Health Observer no. 17. Geneva: World Health Organization; 2016.

3. Ji YB, Lee C. Data envelopment analysis. Stata J. 2010;10(2):267–80.

4. WHO Global Health Estimates 2016 Summary Tables. Estimated DALYs ('000) by cause, sex and WHO Member State (1), 2012. Available at: https://www.who.int/healthinfo/global_burden_disease/GHE2016_Deaths_WBInc_2000_2016.xls

5. Scheffler RM, Liu JX, Kinfu Y, Dal Poz MR. Forecasting the global shortage of physicians: an economic-and needs-based approach. Bull World Health Organ, 2008;86:516–23B.
